# Photographic atlas of phlebotomine sand flies collected in north-west and central east Belize

**DOI:** 10.1186/s13071-026-07499-7

**Published:** 2026-06-21

**Authors:** Ngwa Niba Rawlings, Mark Stephen Bailey, Bruno Leite Rodrigues, José Dilermando Andrade Filho, Sergio Ibáñez-Bernal, Orin Courtenay

**Affiliations:** 1 School of Life Sciences, University of Warwick, Coventry, UK; 2 Department of Environmental Health, DMS, Ministry of Defence, London, UK; 3 Warwick Medical School, University of Warwick, Coventry, UK; 4 Academic Department of Military Medicine, RCDM, Birmingham, UK; 5 Instituto René Rachou, Fundação Oswaldo Cruz (FIOCRUZ), Belo Horizonte, Brazil; 6 Red Ambiente y Sustentabilidad, Instituto de Ecología, A.C. (INECOL), Veracruz, Mexico; 7 Zeeman Institute, University of Warwick, Coventry, UK

**Keywords:** Phlebotomine sand flies, Pictorial key, Morphology, Spermathecae, Male genitalia, Cibarium, Belize, Leishmaniasis

## Abstract

**Background:**

Morphological identification of phlebotomine sand flies in Belize has historically relied on greyscale illustrations and taxonomic keys, which can limit identification accuracy and accessibility for non-specialists. Building on our recent investigation of the spatiotemporal distribution of sand flies in the country, we provide a coloured pictorial guide to the species collected.

**Methods:**

Sand flies were collected in north-west and central east Belize between 2023 and 2025 and identified morphologically using established taxonomic keys. Representative voucher specimens were deposited in the Coleção de Flebotomíneos (FIOCRUZ/COLFLEB), Belo Horizonte, Brazil.

**Results:**

We present 65 coloured photomicrographs covering 20 species, including 18 previously reported in Belize and two species (*Lutzomyia longipalpis* and *Psathyromyia soccula*) recorded in the country for the first time. The guide highlights key diagnostic characters, including male genitalia, spermathecae, cibarium, and other external morphological features, and integrates both coloured and greyscale images. A dichotomous key to the collected species is also provided.

**Conclusions:**

This pictorial guide complements existing taxonomic resources and aims to improve consistency and accessibility of sand fly identification in Belize. The integration of coloured photomicrographs with conventional morphological keys provides a practical resource to support vector surveillance and future taxonomic work.

**Graphical Abstract:**

**Figure:**
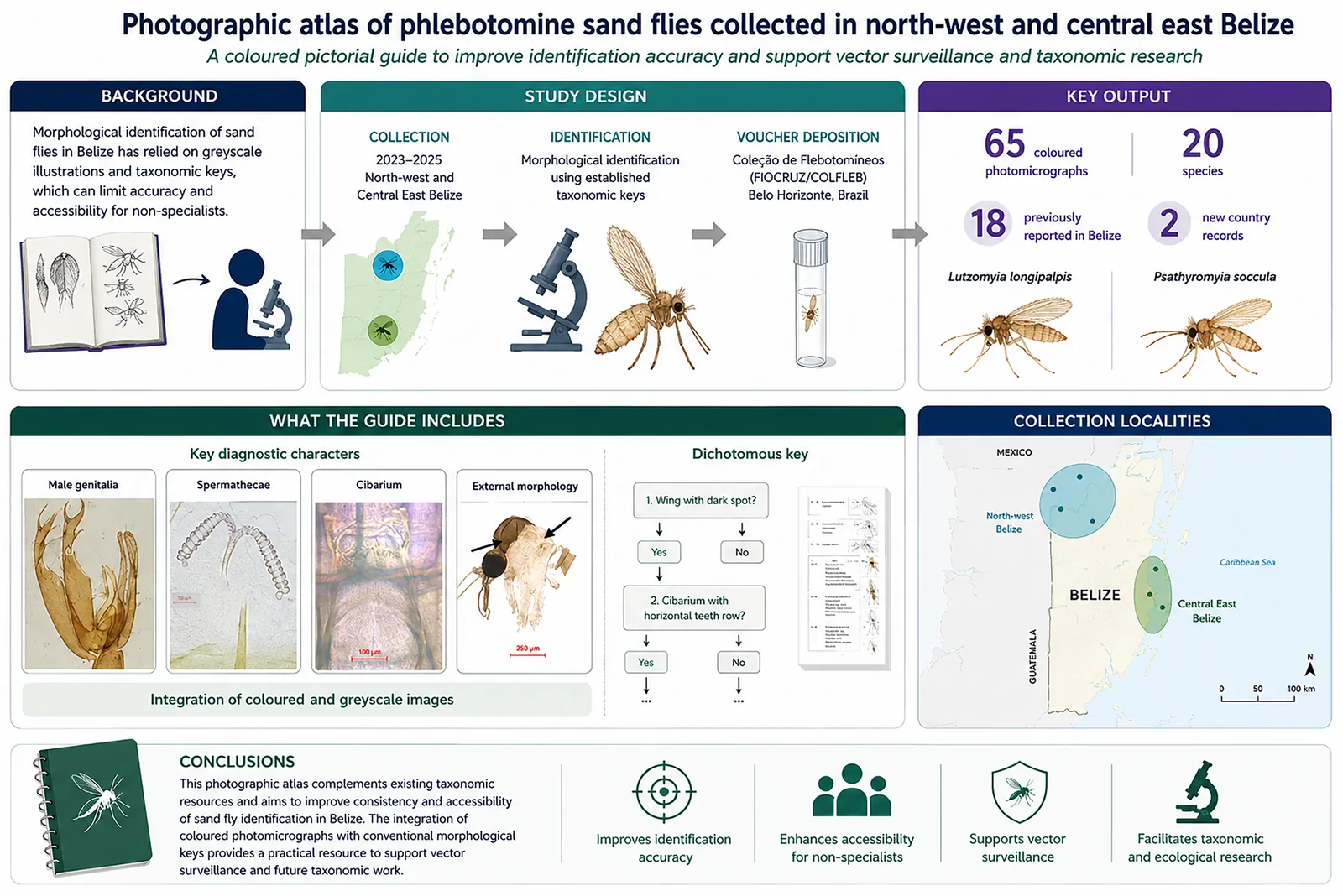

## Background

Phlebotomine sand flies (Diptera: Psychodidae: Phlebotominae) are of major medical importance due to their role as vectors of *Leishmania* parasites (Kinetoplastida: Trypanosomatidae) [1], phleboviruses (Bunyavirales: Phenuiviridae) [2], vesiculoviruses (Mononegavirales: Rhabdoviridae) [3], orbiviruses (Reovirales: Sedoreoviridae; Changuinola virus serogroup) [4], and *Bartonella* spp (Rhizobiales: Bartonellaceae) [5, 6]. In Belize, sand fly fauna has been documented since the mid-twentieth century, with early surveys recording 23 species within *Brumptomyia* and *Lutzomyia* sensu lato [7–17]. Subsequent taxonomic revisions and phylogenetic reclassification expanded the known fauna, including additional species records reported by Ibáñez-Bernal [18]. These taxa are currently distributed across several genera and subtribes under modern classification frameworks [19, 20].

Several species occurring in Belize are recognised or suspected vectors of *Leishmania* spp., including *Bichromomyia olmeca olmeca* (Vargas & Díaz-Nájera, 1959), *Lutzomyia cruciata* (Coquillett, 1907), *Nyssomyia ylephiletor* (Fairchild & Hertig, 1952), *Pintomyia ovallesi* (Ortiz, 1952), *Psathyromyia shannoni* (Dyar, 1929), *Psychodopygus bispinosus* (Fairchild & Hertig, 1951)*,* and *Psychodopygus panamensis* (Shannon, 1926) [7, 9, 10, 15, 16, 21–24]. These species are also implicated in transmission cycles elsewhere in Central and South America [25–27], highlighting the importance of accurate species identification for comparative surveillance and epidemiological investigations.

Early studies in Belize used a wide range of sand fly collection methods, including manual aspiration, human landing catches, light traps, non-human baited traps, sticky traps, and Shannon traps [7–14], and contributed substantially to taxonomic knowledge and species descriptions [18]. Morphological identification traditionally relies on dichotomous keys and greyscale illustrations of diagnostic characters, including male genitalia and female internal structures [13, 14, 16, 34]. While these resources remain fundamental, the absence of coloured pictorial references may limit identification efficiency, particularly for users with limited taxonomic experience.

The present study addresses this gap by providing a coloured pictorial guide to sand fly species collected during recent surveys in Belize. By integrating coloured photomicrographs with established morphological keys [18, 19], this guide aims to improve identification consistency and support ongoing entomological surveillance.

## Methods

The study was conducted in north-west and central-east Belize. Sand flies were collected during the wet seasons of 2023 and 2024 (October to November) and the dry seasons of 2024 and 2025 (February to March and March to April, respectively) across six sites in three districts of north-west and central east Belize (Belize, Orange Walk, and Cayo), all representing peri-urban, pine savanna, and secondary tropical forest environments. Specimens were captured using human landing catches, BG-Sentinel traps with BG-Lure attractant, and CDC light traps. Collected specimens were preserved in 70% ethanol, dissected, and mounted on slides using CMCP-10 high-viscosity mounting fluid (Polysciences, Warrington, USA) for morphological examination under light microscopy. Female specimens were prepared by separating the head and the last three abdominal segments containing the spermatheca, while male specimens were mounted whole.

Species identification followed established taxonomic keys [19], using standard diagnostic terminology for phlebotomine sand flies [29]. Microscopy and identification was done using the Stemi 305 stereomicroscope and Primostar 3 light microscopes (both by Zeiss, Germany) and imaging was performed using an Axiocam 208 colour microscope camera, (Zeiss, Germany). Greyscale illustrations from Ibáñez-Bernal [28] were included with the author and editor’s permission. Scales of greyscale photographs are in millimetres. Key morphological characters used for identification are highlighted in the Results section accompanying each species image, and descriptions of the main diagnostic characters are shown in bold. Abbreviations in the drawings are explained in the list of abbreviations in the supplementary file.

Representative voucher specimens (male and female where available) were deposited in the Coleção de Flebotomíneos (FIOCRUZ/COLFLEB), Belo Horizonte, Minas Gerais, Brazil (Service request: SS04/26). Accession numbers and collection metadata are provided in the Supplementary Material (Table S3).

## Results

Twenty sand fly species were collected, comprising 18 of the 26 species previously recorded in Belize, and two species, *Lutzomyia longipalpis* (Lutz & Neiva, 1912) and *Psathyromyia soccula* (Fairchild & Hertig, 1961), recorded in the country for the first time (voucher accession numbers 92465 and 92451, respectively; Supplementary Table S3). Including these new records, the known phlebotomine sand fly fauna of Belize comprises 28 species.

Following current taxonomic approaches for New World phlebotomine sand flies, the species treated here are assigned to four subtribes and 10 genera.

## Pictorial identification

A consolidated list of all sand fly species recorded in Belize is provided in Supplementary Table S4. The pictorial guide and dichotomous key presented here support the identification of the 20 collected species using morphological characters observable under light microscopy. Key diagnostic characters used to separate higher taxonomic groupings include the configuration of the interocular suture and the arrangement of spines on the gonostyle, alongside other external and internal structures used routinely in adult phlebotomine taxonomy.

## *Bichromomyia olmeca olmeca* (Vargas and Nájera, 1959)

Geographical distribution in Belize: this species has been recorded in the Cayo, Orange Walk, and Toledo Districts [10, 11, 15]. In the present study, specimens were collected in the Cayo District, in Augustine Camp, Merlin, and Neumaria jungle training areas (JTAs).

**Fig. 1:**
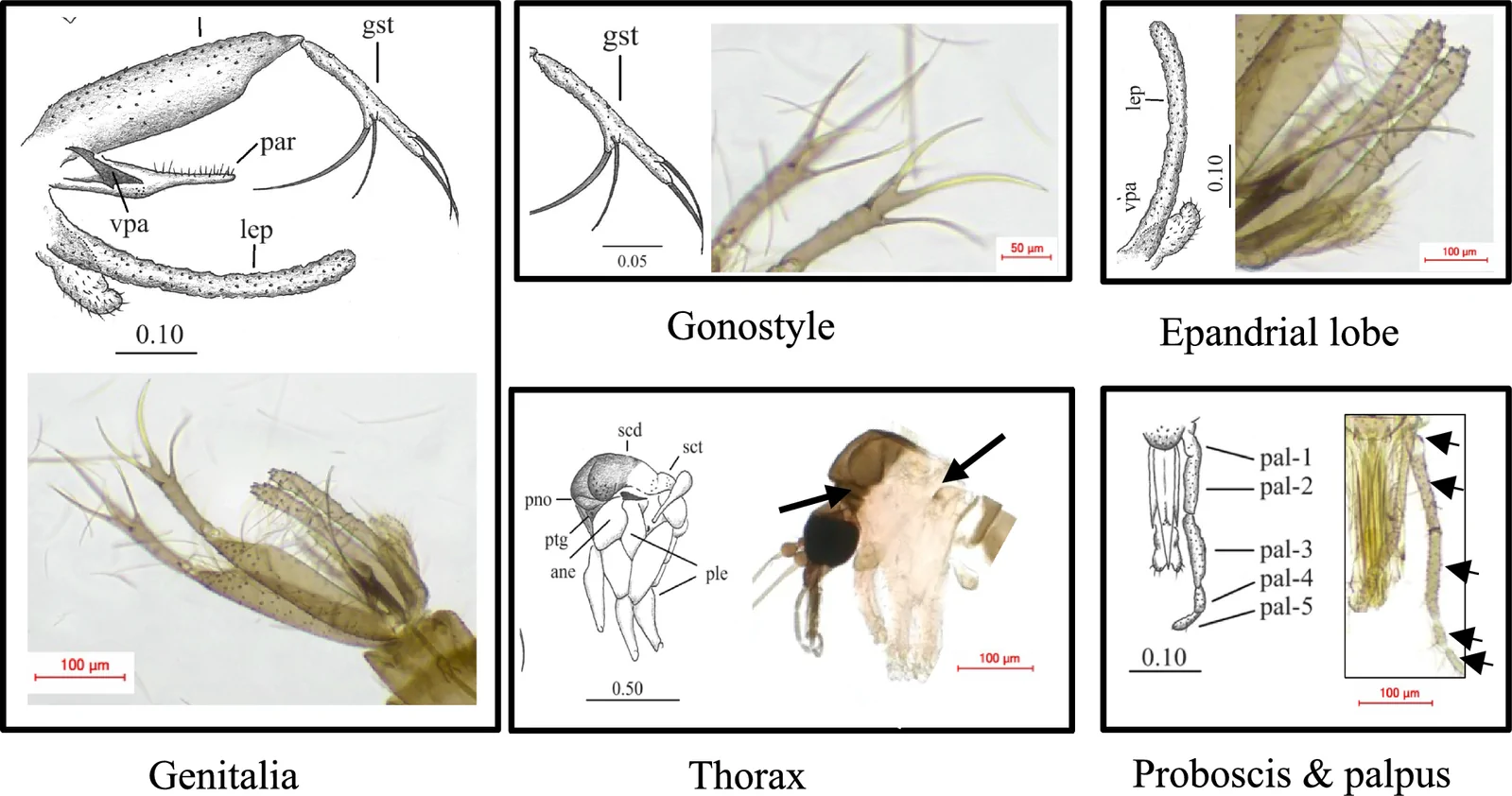
Male of *Bichromomyia olmeca olmeca*

**Male (**Fig. 1**)**

- Palpus 5 is shorter than the combined length of palpi 3 and 4.
- The anterior half of the scutum is dark, like the pronotum, while the posterior half of the scutum and the scutellum are light.
- The gonostyle has a pair of proximal spine-shaped setae at the same level within the apical half of the segment, along with one preapical and one apical spine.
- The epandrial lobe is long and narrow.

Female (Fig. 2)

- The cibarium has prominent anterior teeth arranged in longitudinal rows and about 10–12 long, slender posterior teeth. The pigment patch is funnel shaped.
- The spermatheca has over 10 segments that gradually decrease in size near the individual duct, with a large capitulum about half the diameter of the apical segment. Each individual duct is short before merging with the common duct.

**Fig. 2:**
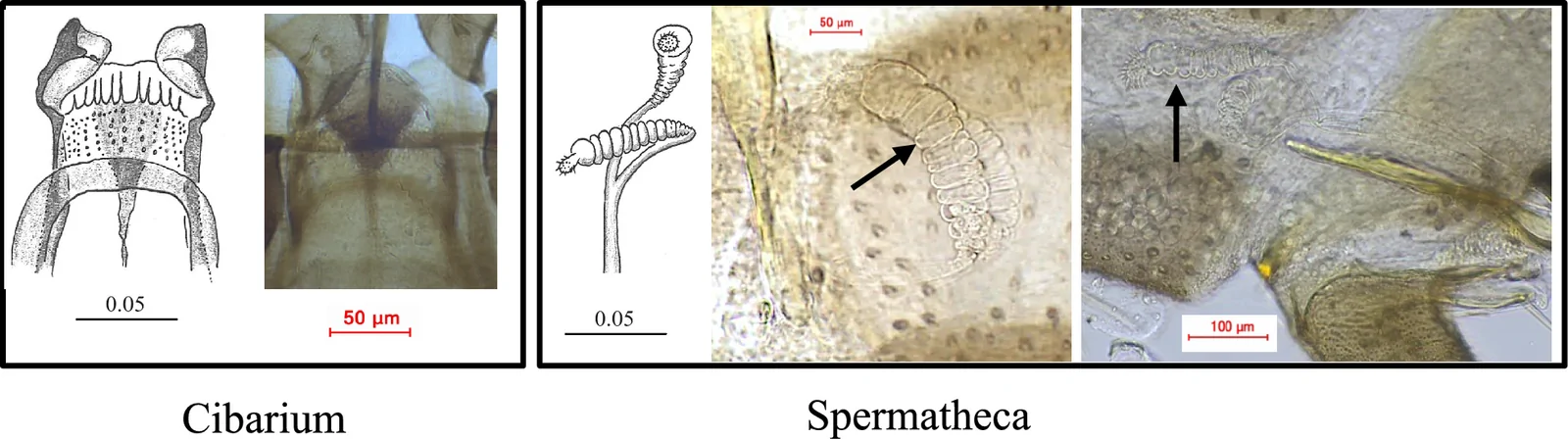
Female of *Bichromomyia olmeca olmeca*

## *Brumptomyia mesai* (Sherlock, 1962)

Geographical distribution in Belize: *Br. mesai* has been recorded in Cayo District (multiple localities) [10, 11, 15] and Toledo District (near Punta Gorda, Blue Creek Preserve), a new district record [18]. In this study, specimens were collected in Cayo District (Augustine Camp, Merlin, and Neumaria JTAs) and in Orange Walk District (Busby Camp JTA).

**Fig. 3:**
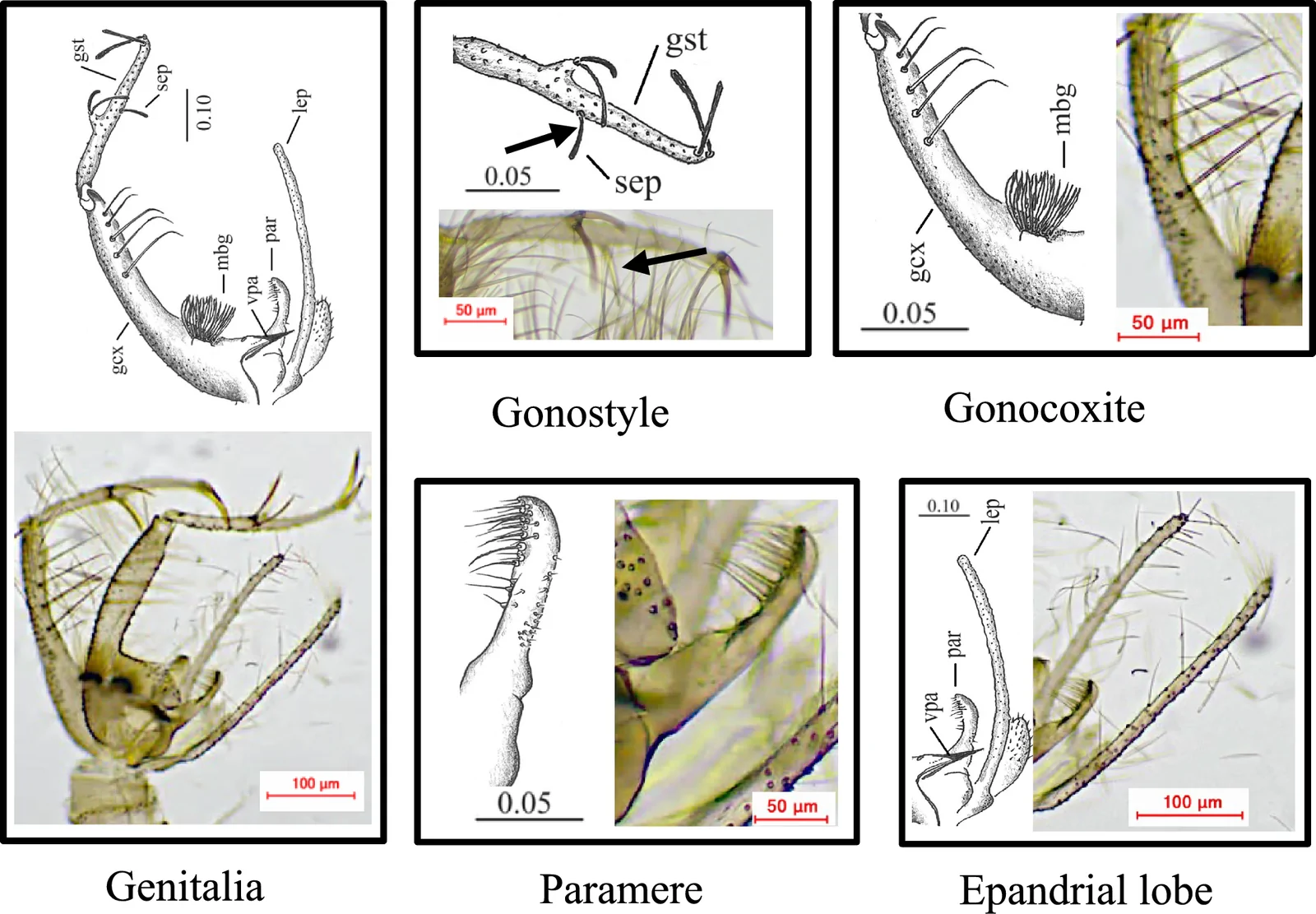
Male of *Brumptomyia mesai*

**Male (**Fig. 3**)**

- The gonostyle has five large spines: a pair of apical spines, a pair of external ones originating from the same tubercle, and an additional isolated internal spine near the middle pair.
- The gonocoxite has 5–6 elongated setae arranged in a longitudinal row, spaced slightly from the apex to the midpoint. A tuft of lanceolate setae is clustered at the base of the gonocoxite.
- The paramere is slender, with a cluster of setae at the distal end.
- The epandrial lobe is long and thin.

Female (Fig. 4)

- Nearly all females of the genus are indistinguishable.
- Partial frontal view of the head shows a complete interocular suture.
- The cibarium has four longitudinal rows of anterior teeth in horizontal position and a dome-like protrusion.
- The spermatheca has a globe-shaped, non-segmented portion near the capitulum, with segments beginning after this part and tapering toward the individual sperm duct.
- Each spermatheca has a very long, coiled individual duct that eventually joins a short common duct.

**Fig. 4:**
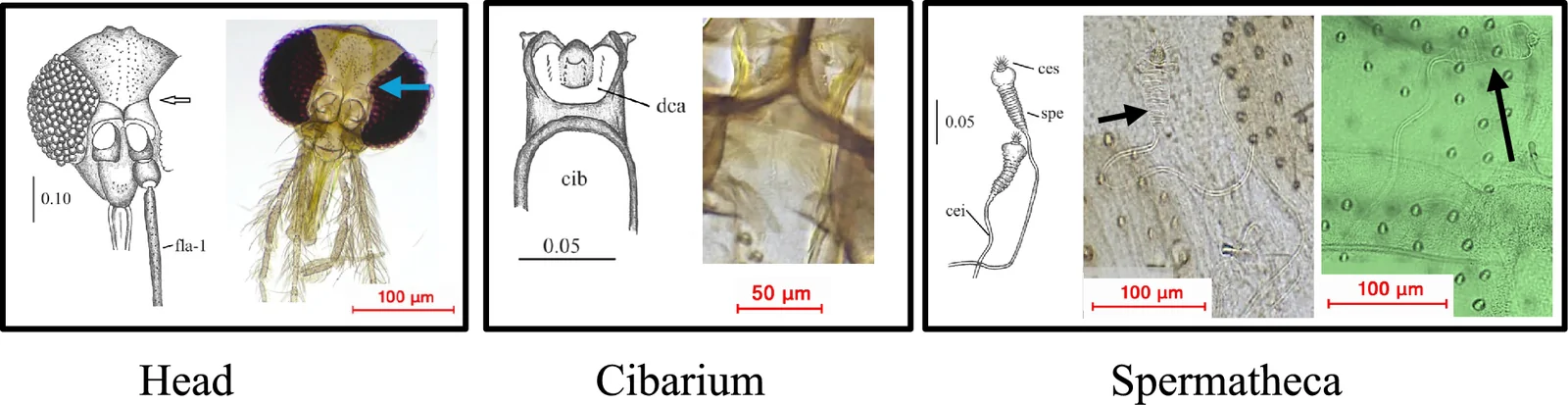
Female of *Brumptomyia mesai*

## *Brumptomyia leopoldoi* (Rodriguez, 1953)

Geographical distribution in Belize: this species has previously been recorded in the Cayo District [10]. In the present study, specimens were collected in the Cayo District (Neumaria JTA).

Male (Fig. 5)

- The gonostyle has five large spines: a pair of apical spines, a pair of external ones originating from the same tubercle, and an additional isolated internal in the same position as the middle pair.
- The aedeagal ducts are eight times the length of the sperm pump.
- The gonocoxite has a basal tuft with upper setae longer than the inferior ones, inserted in a circular patch but not in a tubercle.
- The gonocoxite has six setae in the apical row.
- The paramere is simple.

**Fig. 5:**
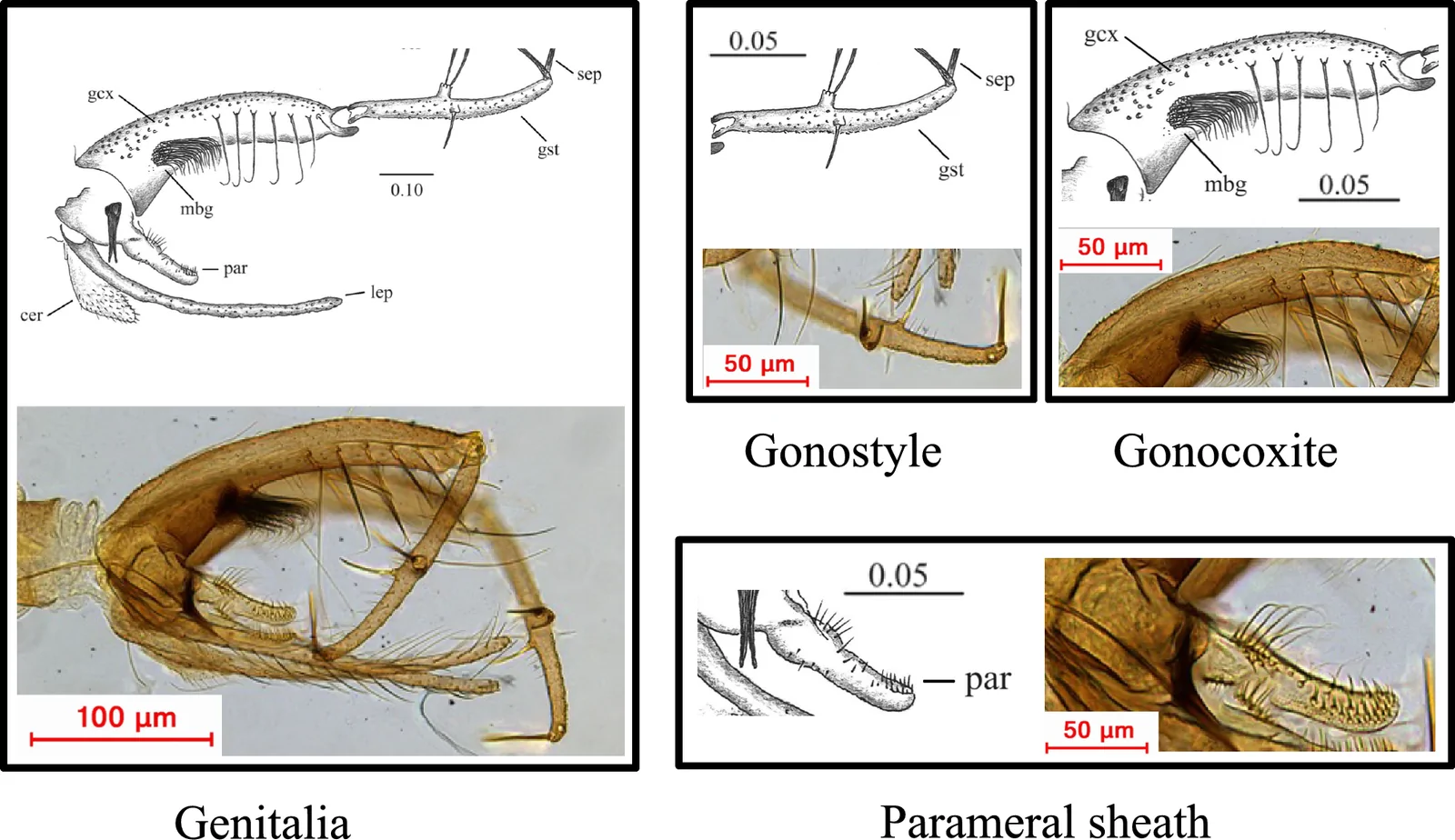
Male of *Brumptomya leopoldoi*

## *Dampfomyia deleoni* (Fairchild & Hertig, 1947)

Geographical distribution in Belize: *Da. deleoni* has been recorded from several localities in Belize, including Cayo District (Chiquibul Road and Roaring River), Orange Walk District (Gallon Jug), Toledo District (near Punta Gorda, Blue Creek Preserve) [9, 10, 15, 18]. In the present study, specimens were collected in Cayo District (Augustine Camp and Neumaria JTAs), Orange Walk District (Busby Camp JTA), and in Belize District (Manatee JTA), representing a new district record.

Male (Fig. 6)

- The gonostyle possesses four prominent, long spines and one pre-apical seta.
- The gonocoxite base features a setal tuft, which is a row of 5–7 simple setae.
- The paramere is elongated and slender in the middle, upward-arcuate, with a ventral angular protuberance at the distal 0.33 of its length.
- The epandrial lobe inflated as long as 7.0 of its length or less than its own diameter.

**Fig. 6:**
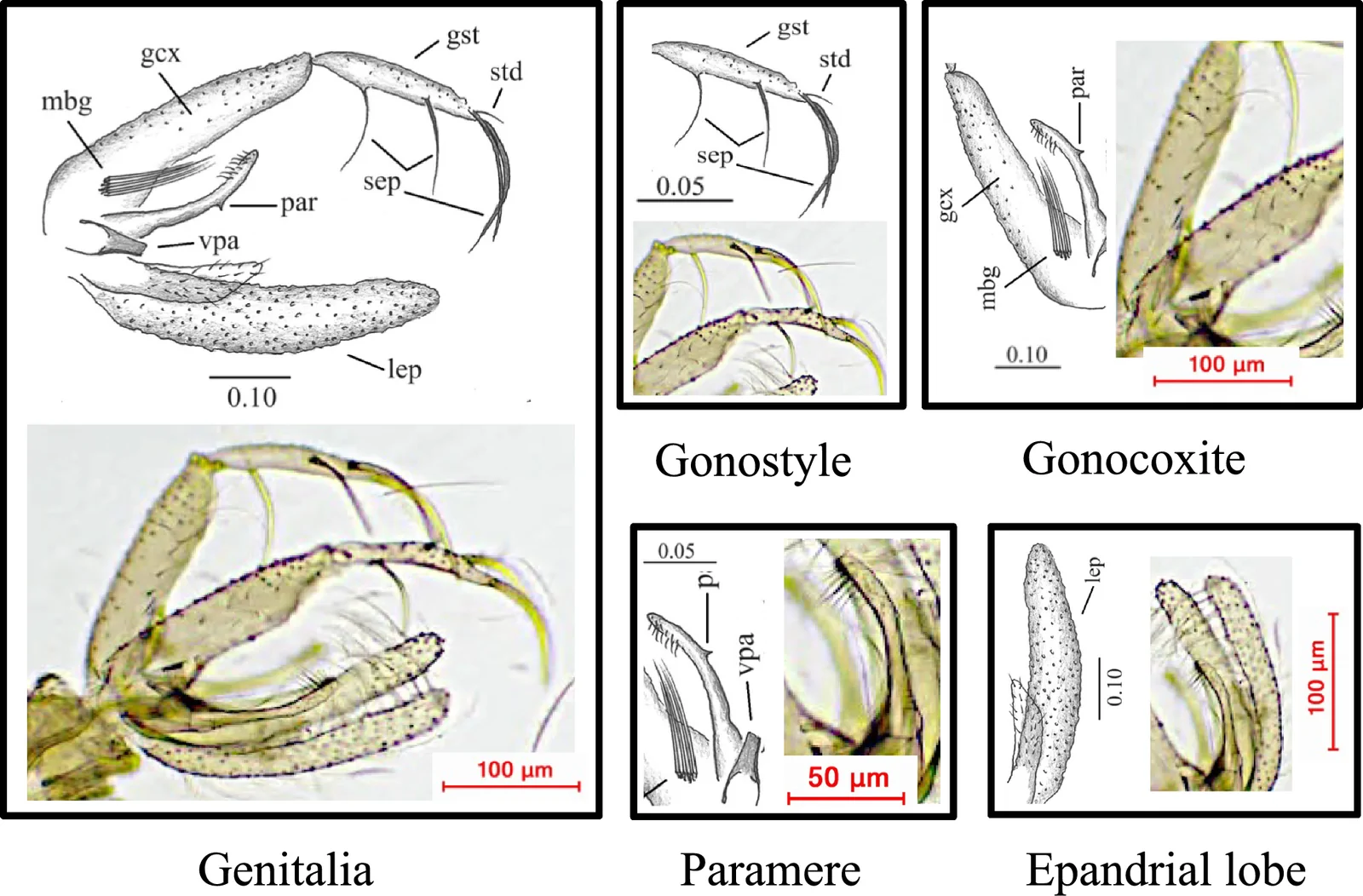
Male of *Dampfomyia. deleoni*

Female (Fig. 7)

- The cibarium contains four posterior teeth arranged like a diadem, with anterior teeth in 1 + 1 diagonal lateral rows.
- The spermatheca has a large, subtly striated, tube-like funnel and features a very long associated membranous sac.

**Fig. 7:**
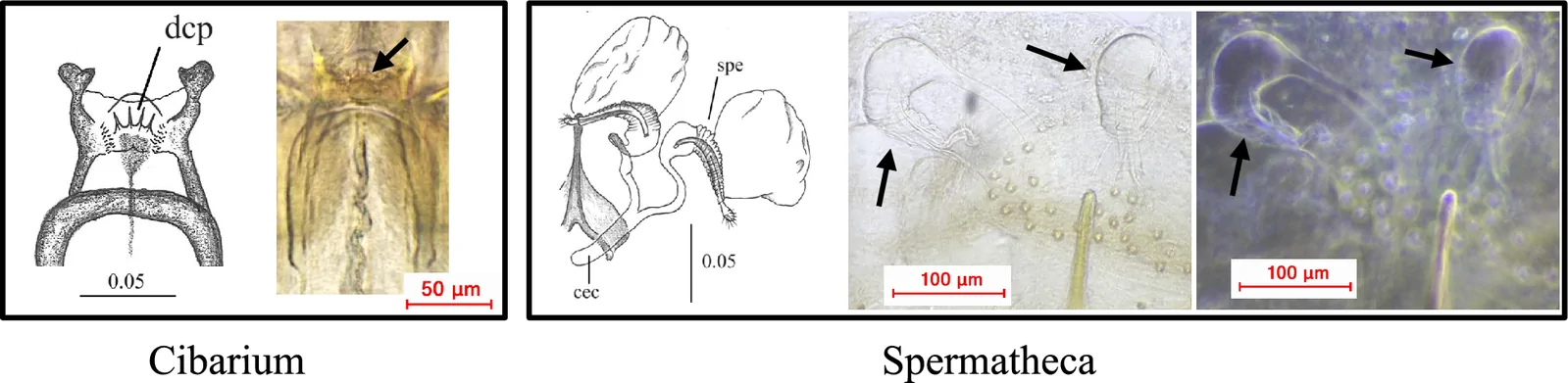
Female of *Dampfomyia deleoni*

## *Dampfomyia disneyi* (*Williams*, 1987)

Geographical distribution in Belize: this species has been recorded in the Cayo District (San Antonio) [13] and the Toledo District (near Punta Gorda, Blue Creek Preserve) [18]. In the present study, specimens were collected in the Cayo District (Augustine Camp and Neumaria JTAs).

Male (Fig. 8)

- The gonostyle has two prominent spines at the distal end, one subtle spine in the basal 0.4 of its length, and a pre-apical seta.
- The gonocoxite has a row of setal tufts at its base.
- The paramere is elongated and slender, with a cluster of setae at its tip.
- The epandrial lobe is shorter than 0.5 times its own greatest diameter.

**Fig. 8:**
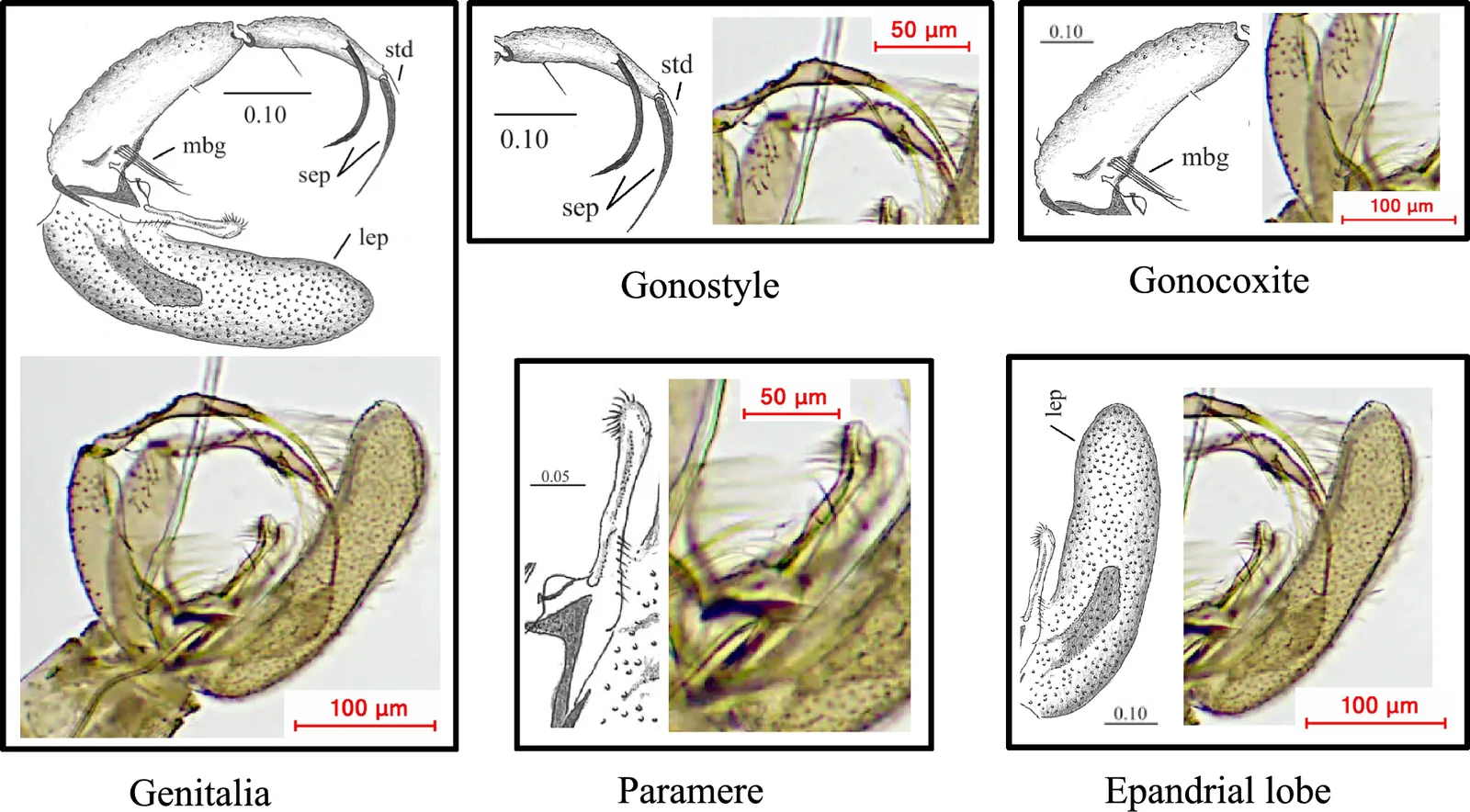
Male of *Dampfomyia disneyi*

## *Dampfomyia aff. permira* (Fairchild & Hertig, 1956)

Geographical distribution in Belize: this species has been recorded in the Cayo District, [10]. In the present study, specimens were collected in the Cayo District (Neumaria JTA).

Male (Fig. 9)

- Palpus 5 is as long as or longer than 0.5 times the length of palpus 3 (see *Ty. triramula*).
- The gonostyle has two prominent spines at the distal end, one subtle basal spine, and a subterminal seta.
- The gonocoxite lacks a setal tuft.
- The paramere has a long and thin dorsal arm and lacks a ventral triangular projection.
- The epandrial lobe is longer than 7.5 times its own diameter and tapers at the end.

**Fig. 9:**
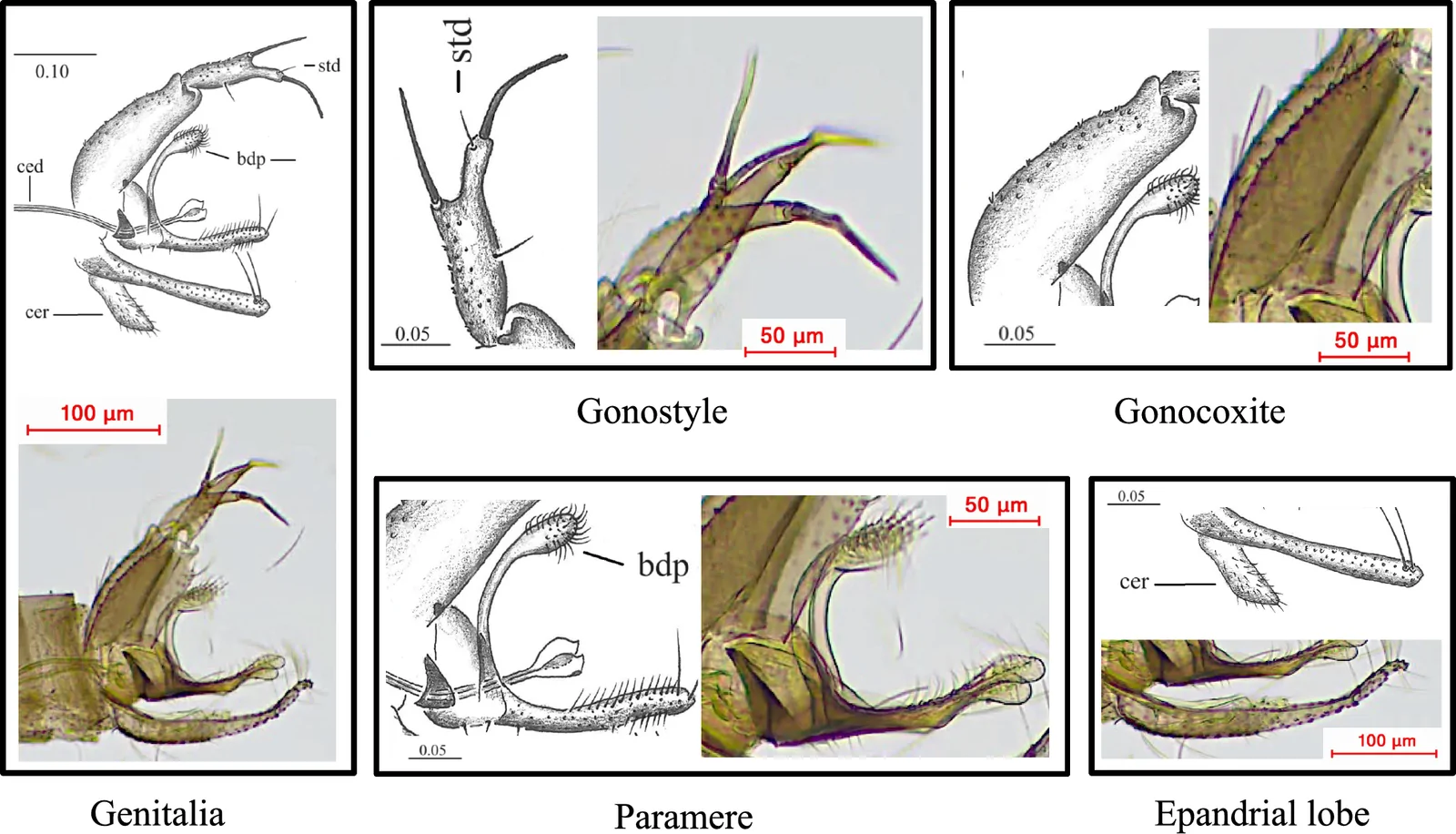
Male of *Dampfomyia aff. permira*

Female (Fig. 10)

- Palpus 5 is longer than 0.5 times the length of palpus 3 (see *Ty. triramula*).
- The cibarium has six short posterior teeth, with anterior teeth nearly invisible. The sclerotised area has a cloud-like shape.
- The spermathecae are rounded, with a complex, lobed appearance at the head (raspberry-like). Individual sperm ducts are extremely short.
- The two rounded spermathecae fuse in the middle into a large common duct.

**Fig. 10:**
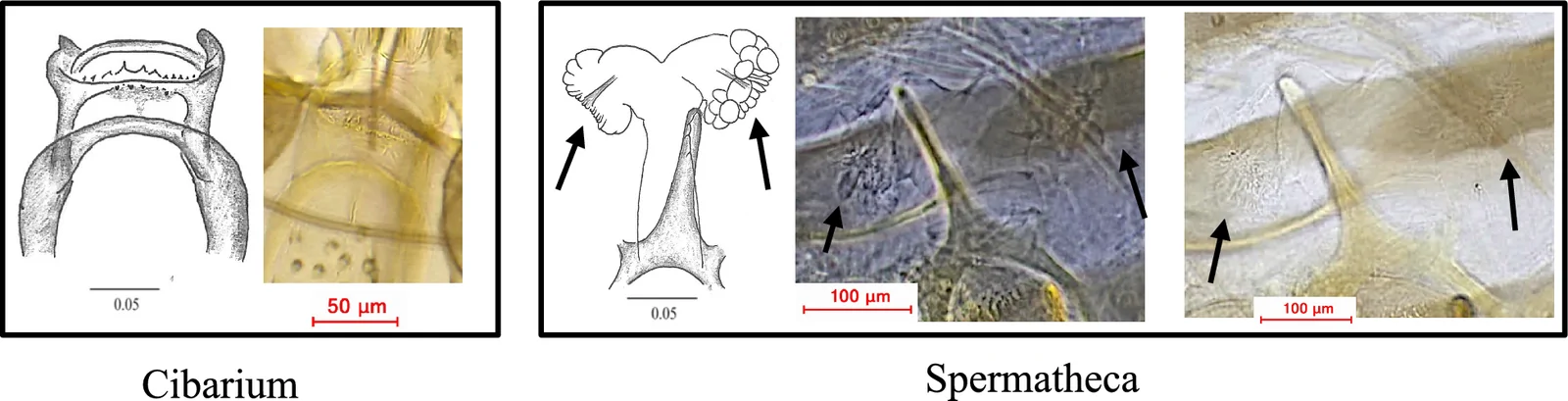
Female of *Dampfomyia aff. permira*

## Comments

The specimens collected and processed in this study from Belize show some differences compared to the illustrations of *Da. permira*. Regarding male terminalia, the ventral branch of our specimens seems to have a pronounced elbow for both parameres (i.e., it does not seem to be a case of distortion due to the mounting position), while illustrations show a more finger-like paramere and uniform in its width. In the same sense, the female spermathecae of this study seem to indicate that the common duct may have collapsed or wrinkled, so that it appears shorter and wider than it actually would be if it were stretched, as in the illustrations. Then, it is possible that our specimens correspond to a distinct sand fly species, which may need a formal description. However, the analysis of a larger number of specimens is necessary to validate these observations, and in this study, it was not possible to compare our specimens with those from the *Da. permira* type series (type locality: Mexico), hampering a comparative analysis. Therefore, we list this *Dampfomyia* species as *affinis* to *Da. permira*, indicating the close morphological relationship among them.

## *Lutzomyia cruciata* (Coquillett, 1907)

Geographical distribution in Belize: this species has been recorded in the Cayo District (San Antonio) [9–11, 15] and the Toledo District (near Punta Gorda, Blue Creek Preserve) [18]. In the present study, specimens were collected in Cayo District (Augustine Camp and Neumaria JTAs) and Belize District (Manatee JTA), representing a new district record.

Male (Fig. 11)

- Palpus 5 is longer than the combined length of palpi 3 and 4 (see Ty. triramula).
- ▪The scutum, pronotum, and paratergite are dark, while the rest of the pleura is pale.
- ▪The gonostyle is as long as or longer than 0.5 times the length of the gonocoxite and has four spines: two externals, one internal, and one apical, with the internal one near the lower external.
- ▪A tuft of setal hairs is located at the middle of the gonocoxite, with alveoli arranged in a raspberry-like pattern.
- ▪The paramere is elongated and slender, with setal hairs at its tip and a dark, prominent parameral sheath at the base.
- ▪The epandrial lobes are long and slightly curved.

**Fig. 11:**
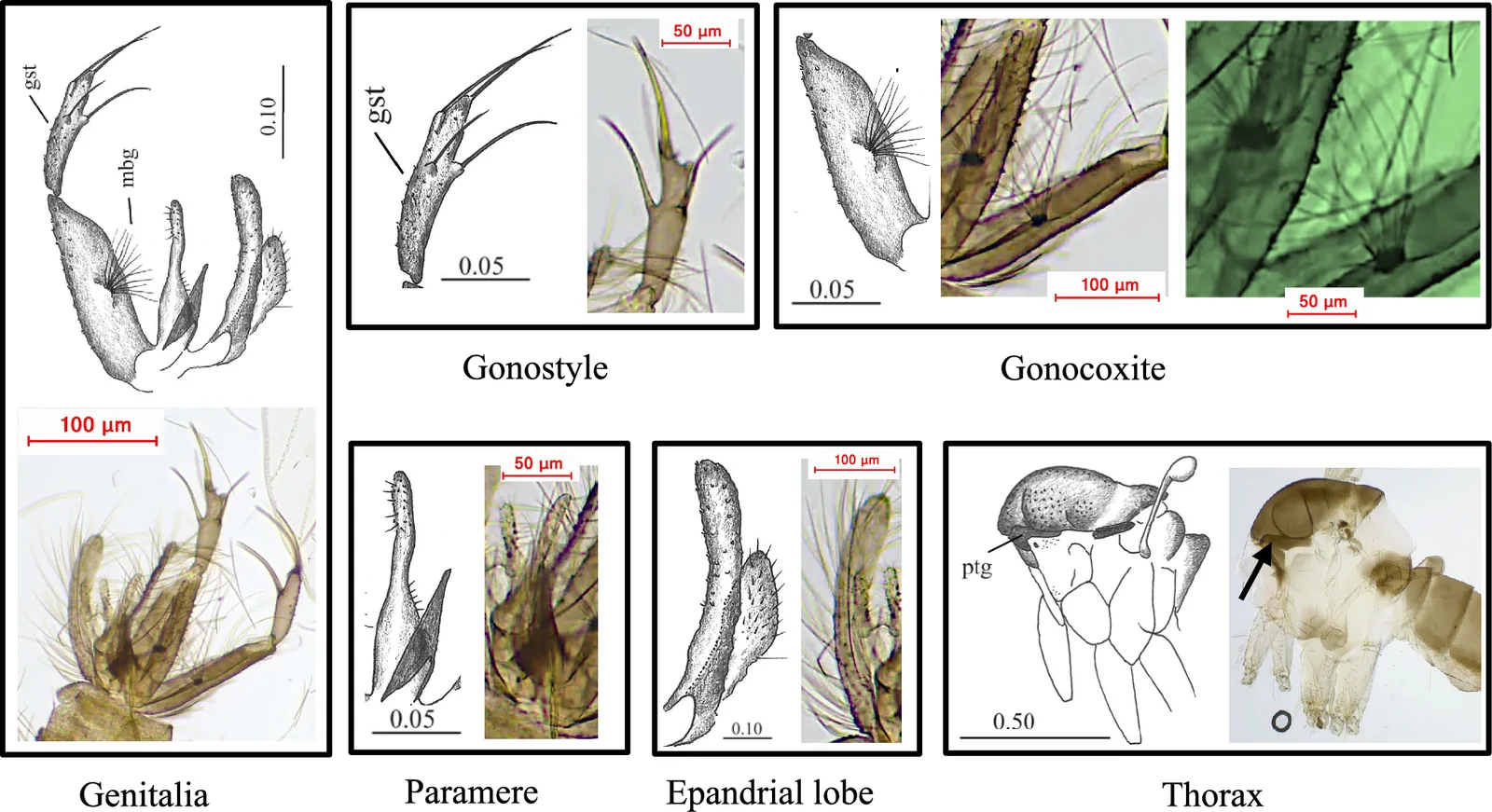
Male of *Lutzomyia cruciata*

Female (Fig. 12)

- Palpus 5 is longer than the combined length of palpi 3 and 4 (see Ty. triramula).
- ▪The cibarium has prominent posterior 4 teeth that are slightly spaced apart, while the anterior teeth are aligned in a row, overlapping the top of the pigment patch.
- ▪The spermathecae are piriform, with a round, bulb-like head and a long, coiled, faintly segmented base that narrows toward the base. The head is invaginated into the body.

**Fig. 12:**
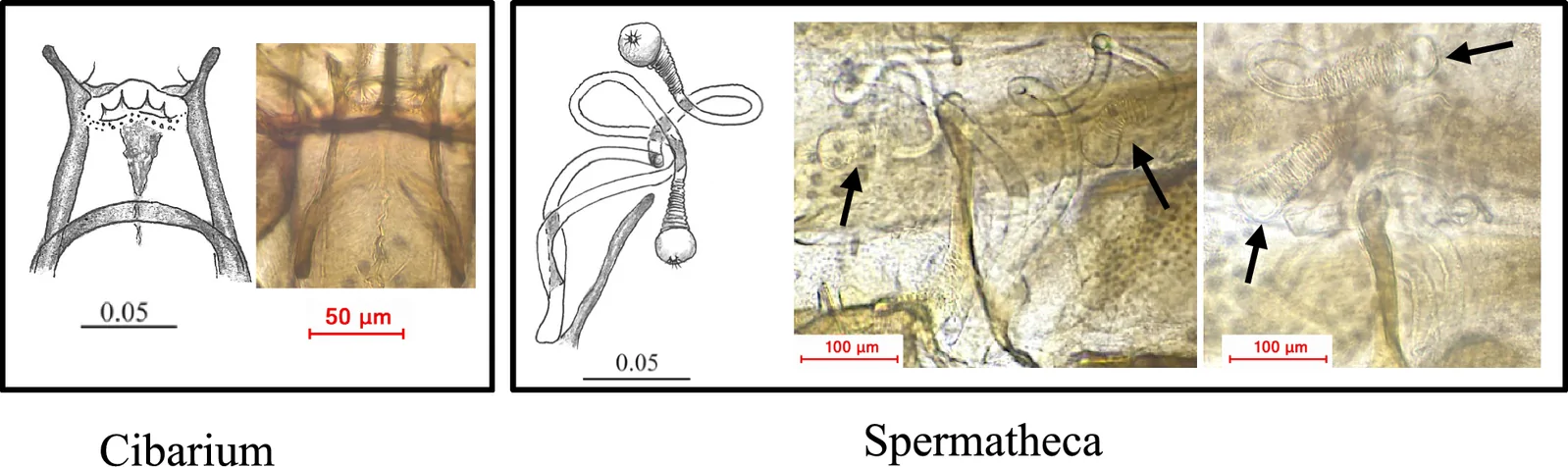
Female of *Lutzomyia cruciata*

## *Lutzomyia longipalpis* (Lutz and Neiva, 1912)

Geographical distribution in Belize: this species has not previously been recorded in Belize. In the present study, a specimen was collected in the Merlin JTA, Cayo District, representing a new country record.

Male (Fig. 13)

- Gonostyle with for spines and present a pre-apical seta.
- A tuft of setal hair is located at the base of the gonocoxite.
- Paramere with 2 curved hook-shaped setae originating from a dorsal region.

**Fig. 13:**
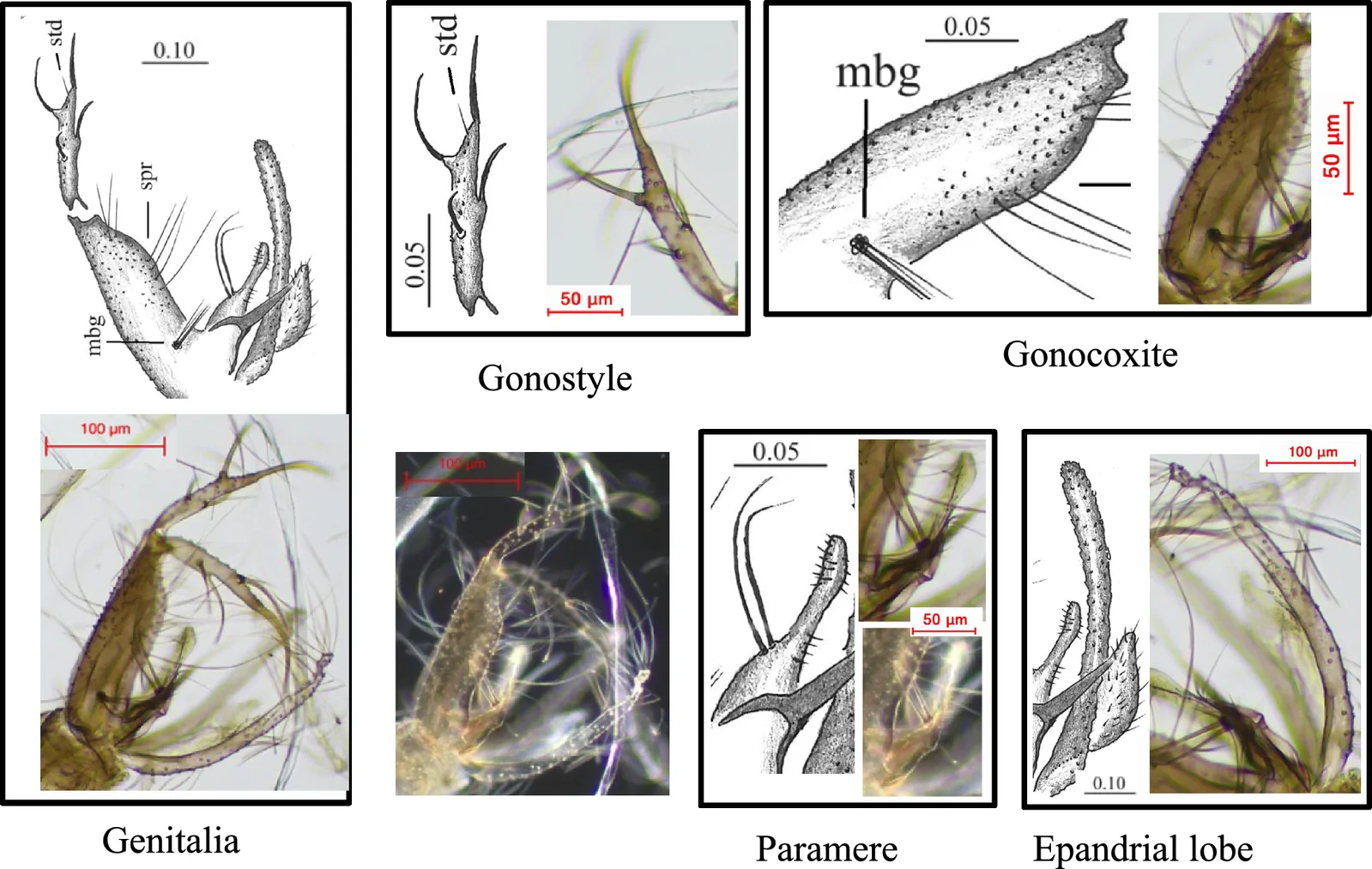
Male of *Lutzomyia longipalpis*

## *Micropygomyia trinidadensis* (Newstead, 1922)

Geographical distribution in Belize: this species has been recorded in the Cayo District [10]. In the present study, specimens were collected in the Cayo District (Augustine Camp and Neumaria JTAs).

Male (Fig. 14)

- Palpus 5 is longer than 0.5 times the length of palpus 3 (see *Ty. triramula*).
  o▪ Wing venation with a positive δ index, characterised by vein R₂ + ₃ extending beyond the level of R₁, resulting in a clearly positive *δ* value.
- The gonostyle has five prominent spines: two apical, one upper external, one lower external, and the internal one located near the lower external one.
- The gonocoxite has persistent setae.
- The epandrial lobe is thin, and the paramere is simple.

**Fig. 14:**
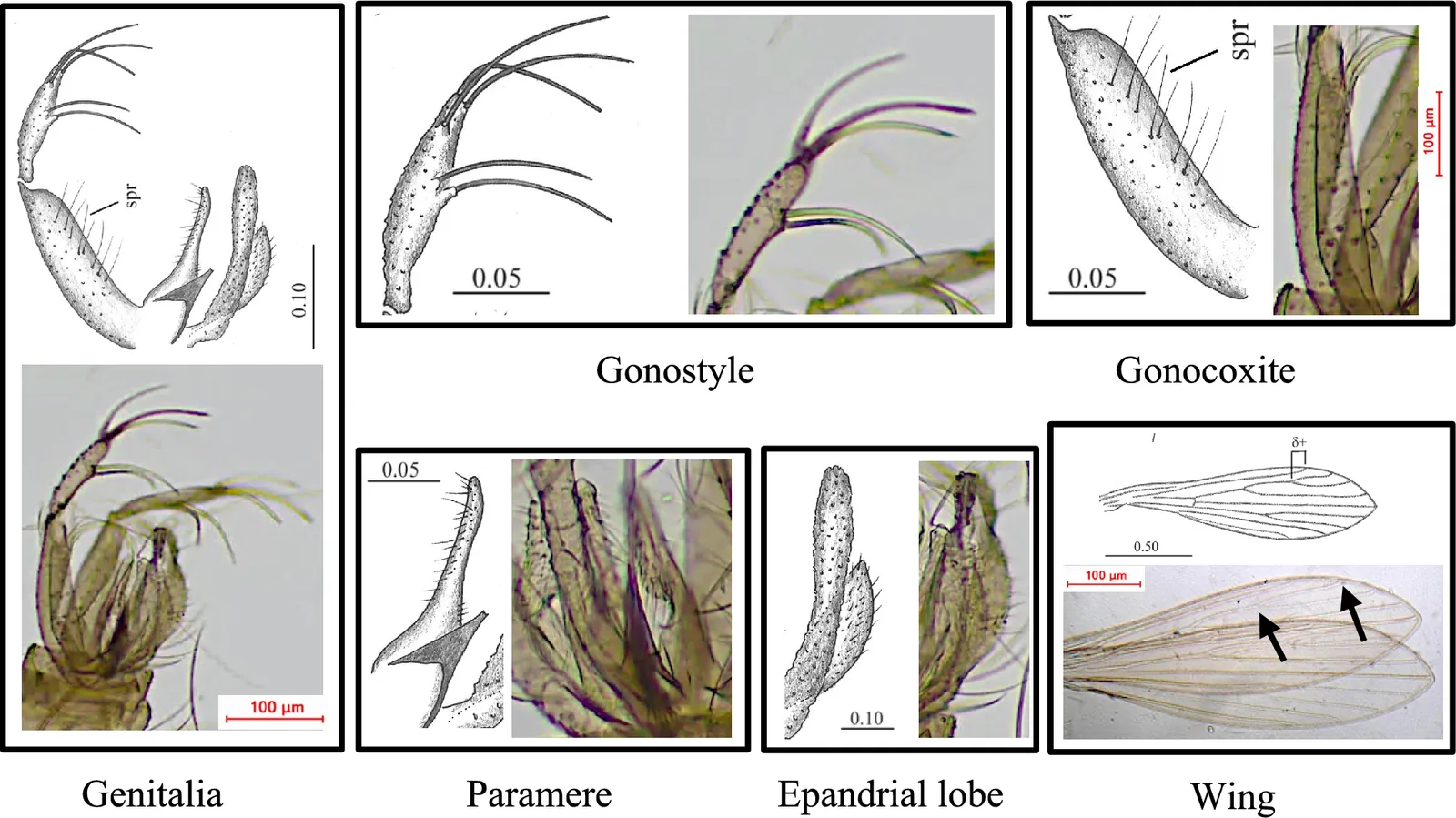
Male of *Micropygomyia trinidadensis*

Female (Fig. 15)

- Palpus 5 is longer than 0.5 times the length of palpus 3 (see *Ty. triramula*).
- Several rows of spines are present in the pharyngeal armature, exhibiting a flame-like appearance and occupying one-third of the pharynx.
- The four posterior teeth are paired on either side of the cibarium and separated by an arch, with no visible anterior teeth.
- The spermathecae are long, slender, sausage-shaped tubes with a smooth wall that narrows toward the individual duct. The individual ducts coil to about twice the length of the spermathecae and join into a common duct, which is approximately 0.75 times the length of the spermathecae.

**Fig. 15:**
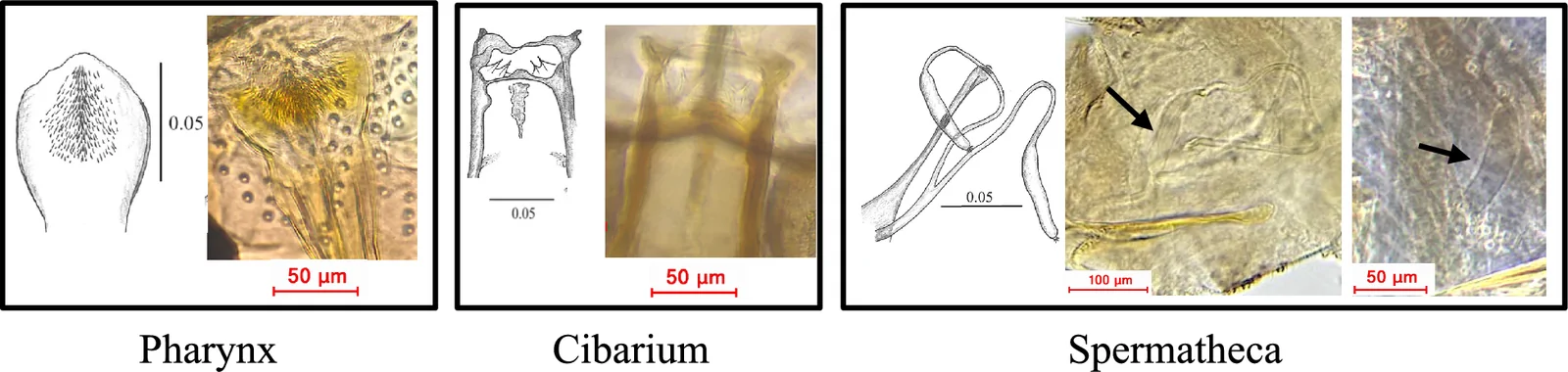
Female of *Micropygomyia trinidadensis*

## *Nyssomyia ylephiletor* (Fairchild and Hertig, 1952)

Geographical distribution in Belize: this species has been recorded in Cayo District and Orange Walk District [9–11, 15], as well as in Toledo District (near Punta Gorda, Blue Creek Preserve [18]. In the present study, specimens were collected in the Cayo District (Augustine Camp and Neumaria JTAs).

Male (Fig. 16)

- Palpus 5 is shorter than the combined length of palpi 3 and 4 (see *Bi. olmeca olmeca*).
- The scutum is dark, while the scutellum is somewhat paler (see *Bi. olmeca olmeca*).
- The gonostyle has four spines: one apical, one upper external, one lower external, and the internal one located near to the lower external.
- The gonocoxite has fewer than five isolated setae in the apical region.
- The paramere is slender and elongated, with fine, bristle-like setae.
- The epandrial lobes are long.

**Fig. 16:**
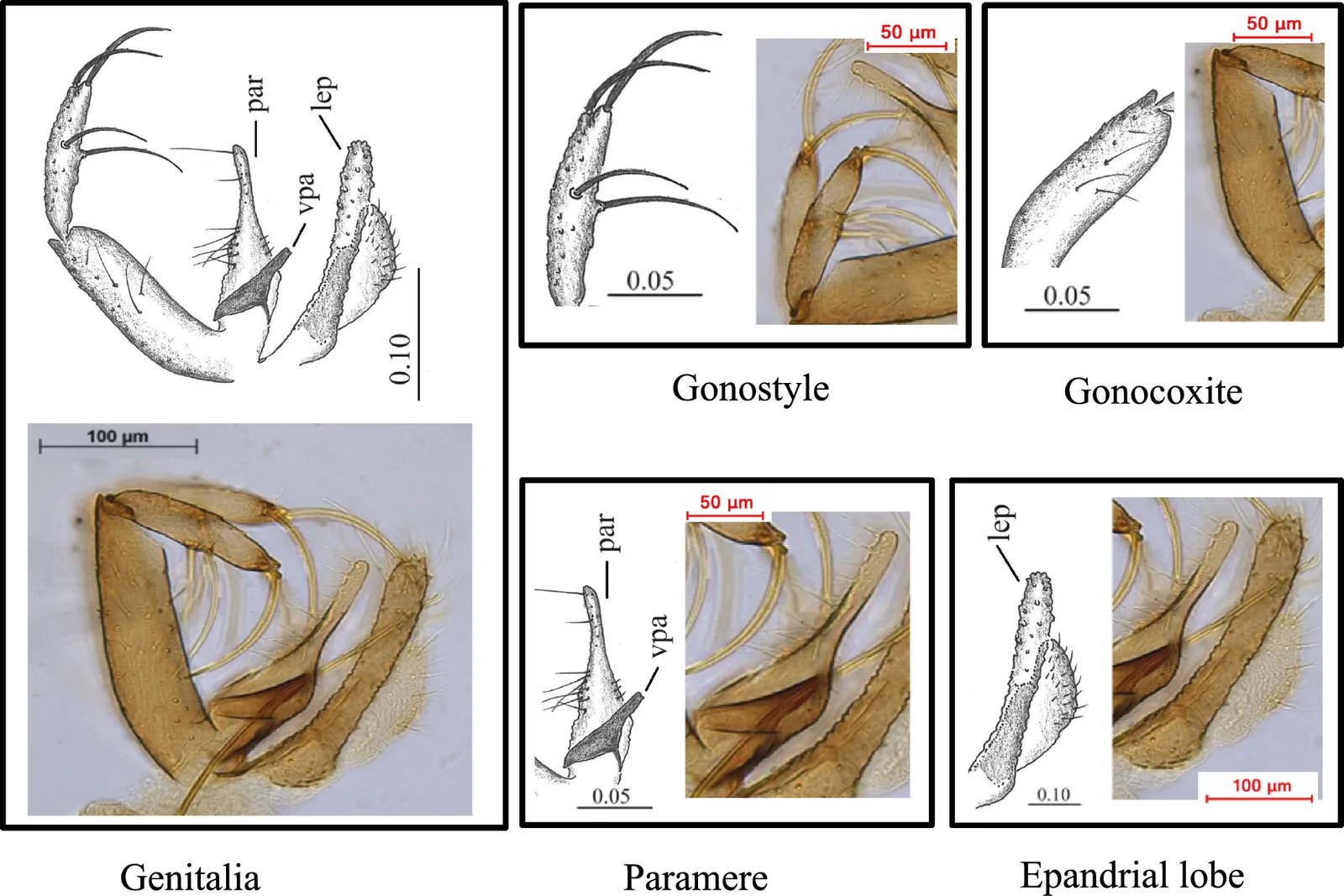
Male of *Nyssomyia ylephiletor*

Female (Fig. 17)

- About 12 elongated and prominent posterior teeth are positioned above various rows of anterior teeth in the cibarium.
- The pigment patch is funnel-shaped, with a wide opening that narrows into a thin stem.
- Each spermatheca has a large, round head adorned with a dense tuft of setae radiating outward.
- The spermathecae are ringed and slightly curved, with a beaded appearance.
- The individual ducts are short, approximately the length of the spermatheca, slender, and converge into a very short common duct, which is about 0.25 times the length of the spermatheca.

**Fig. 17:**
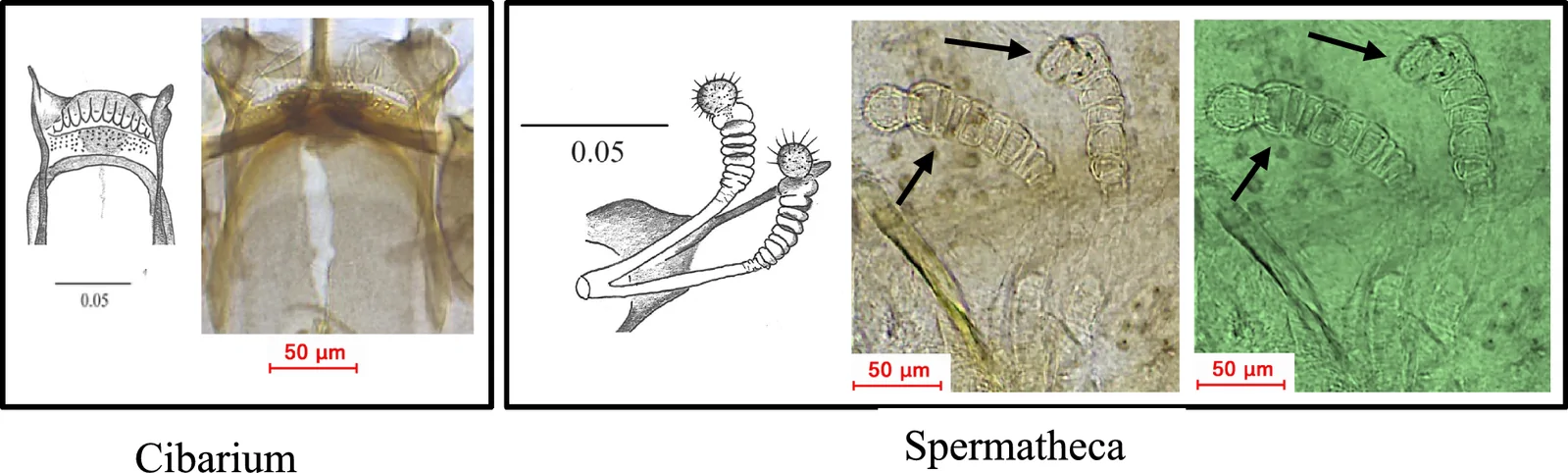
Female of *Nyssomyia ylephiletor*

## *Pintomyia ovallesi* (Ortiz, 1952)

Geographical distribution in Belize: this species has been recorded in the Cayo District [8–11, 15] and the Toledo District (near Punta Gorda, Blue Creek Preserve [18]. In the present study, specimens were collected in the Cayo District (Merlin and Neumaria JTAs).

Male (Fig. 18)

- Palpus 5 is longer than 0.5 times the length of palpus 3 (see *Ty. triramula*).
- The gonostyle has four spines: one apical, one upper external, one lower external, and the internal one located near the lower external. It is shorter than 0.4 times the length of the gonocoxite.
- The base of the gonocoxite has a setal tuft, consisting of a row of five setae.
- The paramere is elongated and slender, with a cluster of setae at its tip.
- The epandrial lobes are long.

**Fig. 18:**
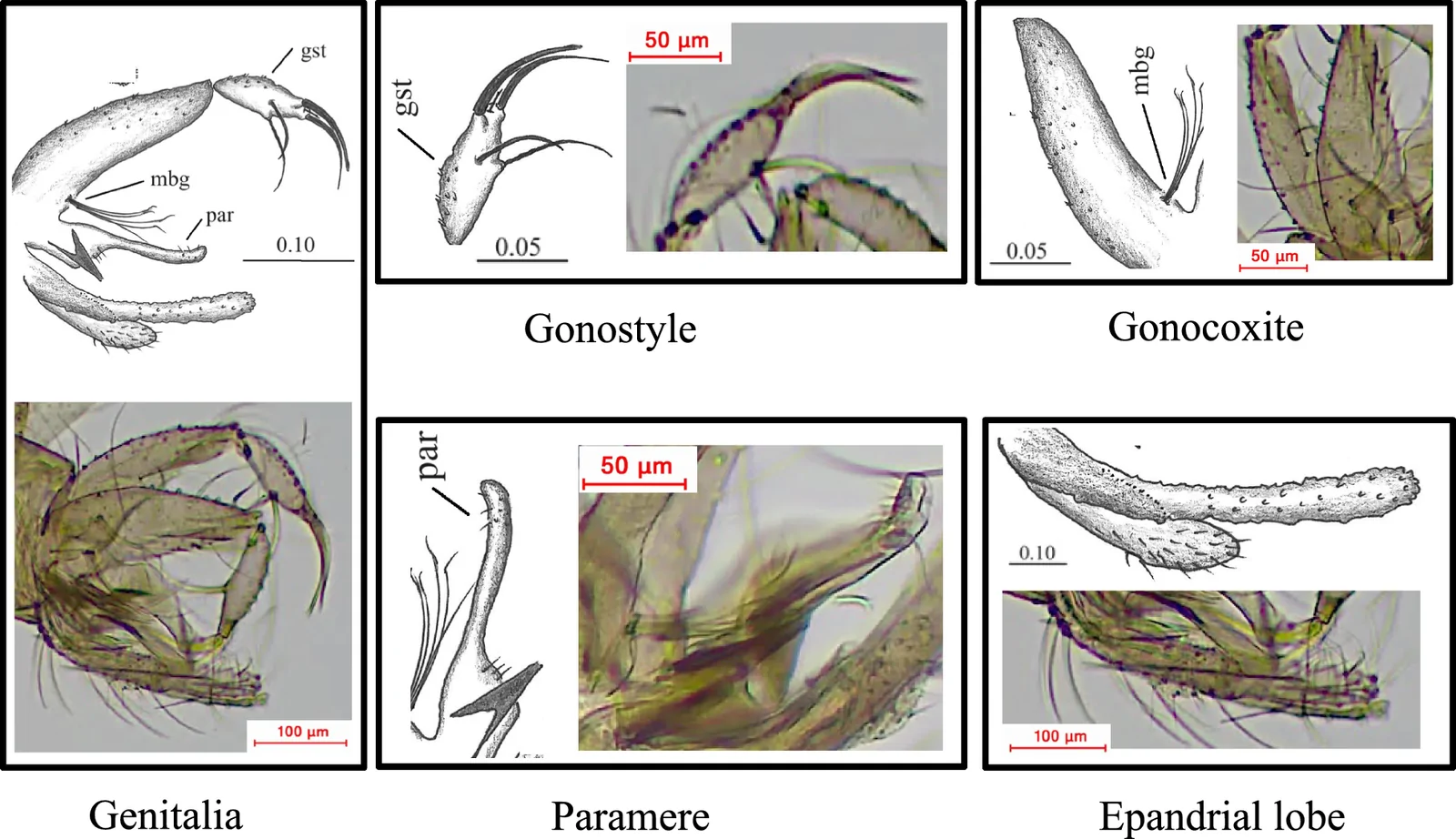
Male of *Pintomyia ovallesi*

Female (Fig. 19)

- Several rows of elongated spicules are present in the pharyngeal armature.
- Four posterior teeth are positioned above a row of anterior teeth in the cibarium.
- Each spermatheca has prominent segmentation, giving it a ringed appearance, with a head that appears to invaginate into the body.
- The spermatheca narrows toward the base or is constricted near the middle, where it transitions into the individual ducts.
- The short individual ducts connect to the common duct.

**Fig. 19:**
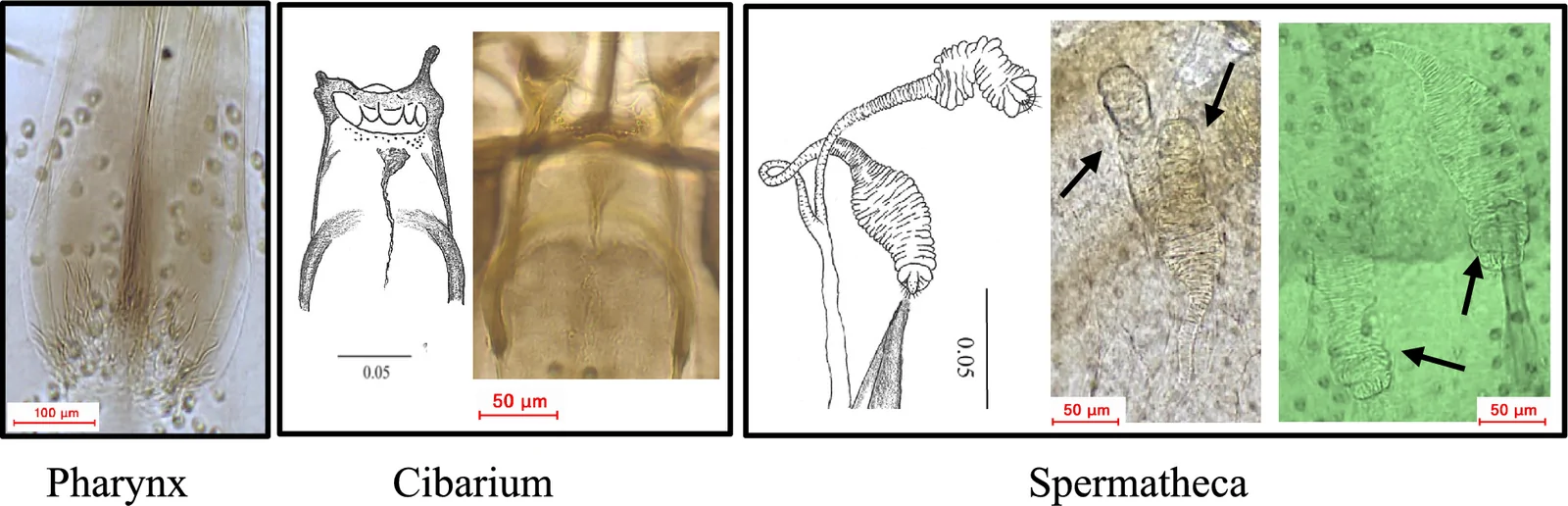
Female of *Pintomyia ovallesi*

## *Pintomyia serrana* (Damasceno and Arouck, 1949)

Geographical distribution in Belize: this species has previously been recorded only in the Toledo District (near Punta Gorda, Blue Creek Preserve) [18]. In the present study, specimens were collected in Cayo District (Augustine Camp and Neumaria JTAs), representing a new district record.

Female (Fig. 20)

- A row of four posterior teeth is evenly spaced and aligned above a row of anterior teeth.
- Each spermatheca has prominent segmentation, giving it a ringed appearance. The spermatheca narrows toward the base, where it connects with thick individual ducts.
- The individual ducts are short and connect to the common duct, which is three times the length of the spermatheca.
- The head of the spermatheca extends visibly through one-third of the body.

**Fig. 20:**
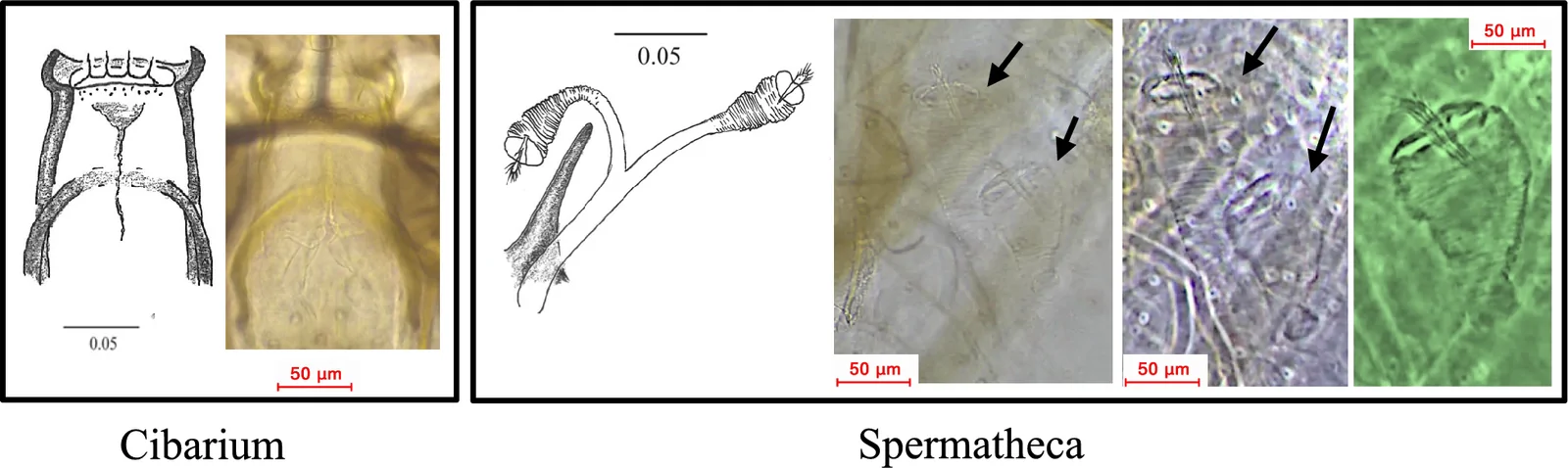
Female of *Pintomyia serrana*

## *Psathyromyia carpenteri* (Fairchild & Hertig, 1953)

Geographical distribution in Belize: this species has been recorded in the Cayo district [10, 15] and the Toledo District (near Punta Gorda, Blue Creek Preserve [18]. In the present study, specimens were collected in Cayo District (Augustine Camp, Busby Camp, Merlin, and Neumaria JTAs) and Belize District (Manatee JTA), representing a new district record.

Male (Fig. 21)

- The apex of vein R1 in the wing is approximately at the midpoint of vein R2.
- The elongated gonostyle bears four prominent spines: one apical spine, an upper external spine implanted at the beginning of the apical third, a lower external spine situated slightly before the middle of the gonostyle, and an inner spine positioned almost apically.
- The gonocoxite is large, with more than 20 sparse setae at the centre.
- The paramere is long, slender, and slightly curved upward.
- The epandrial lobes are long and slightly curved.

**Fig. 21:**
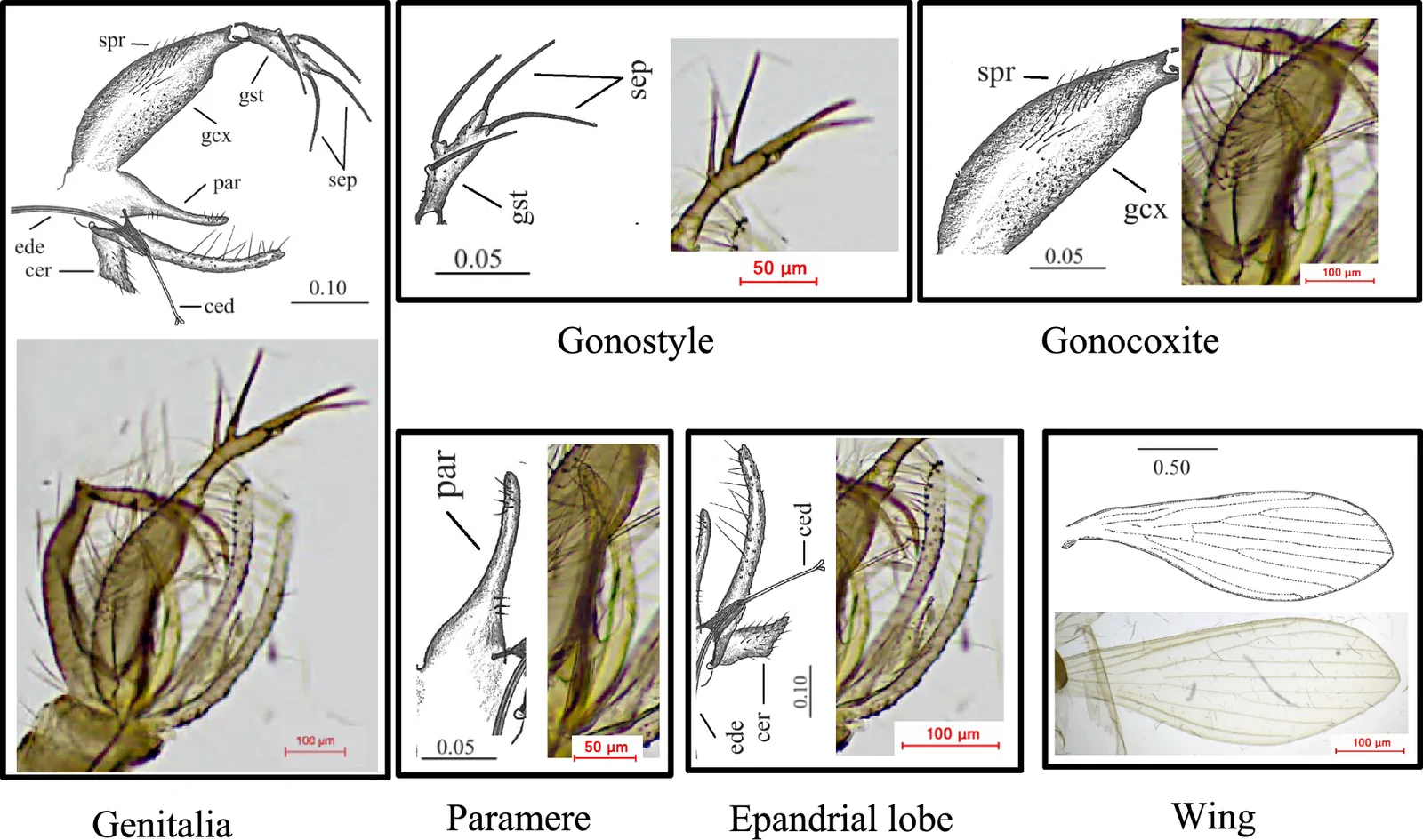
Male of *Psathyromyia carpenteri*

Female (Fig. 22)

- Cibarium has anterior teeth arranged in two irregular rows, with all teeth being small, and the sclerotised area is incomplete.
- Ascoids have a short proximal spur (see *Pa. shannoni*).
- Each spermatheca is pear-shaped, with a wide neck at its junction with the individual duct.

**Fig. 22:**
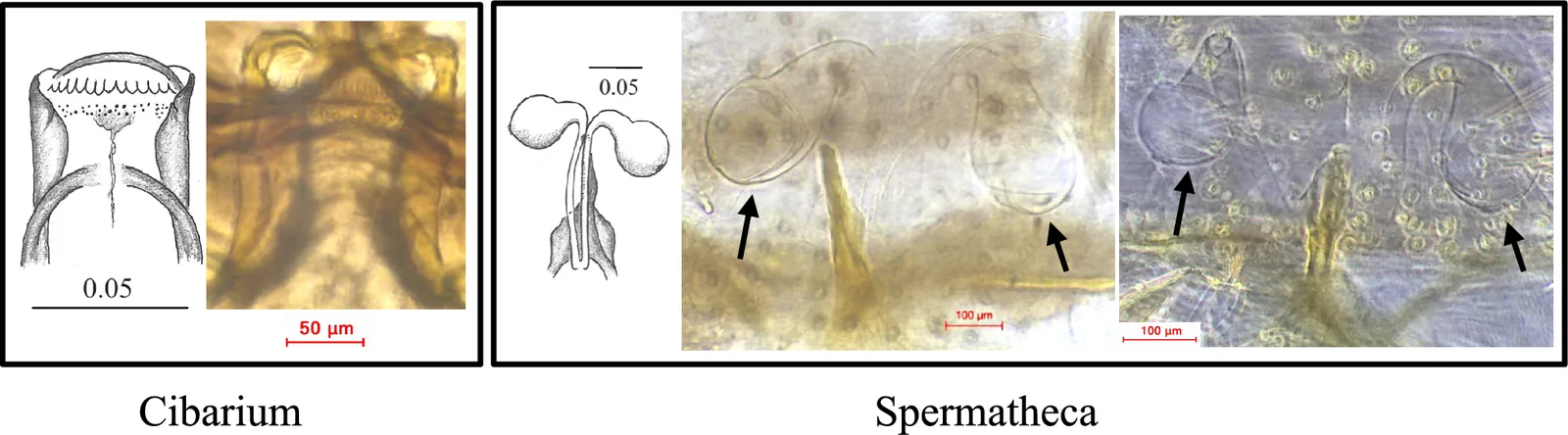
Female of *Psathyromyia carpenteri*

## *Psathyromyia shannoni* (Dyar, 1929)

Geographical distribution in Belize: this species has been recorded in Cayo District, Orange Walk District, and Toledo District [9, 10, 15, 18]. In the present study, specimens were collected in the Cayo District (Merlin and Neumaria JTAs) and the Orange Walk District (Busby Camp JTA).

Male (Fig. 23)

- Ascoids have a long proximal spur.
- The gonostyle has four spines: one apical, one upper external, one lower external, and the internal one located near the lower external.
- The gonocoxite lacks a setal tuft.
- The paramere is slender and elongated.
- The epandrial lobes are long and slightly curved.

**Fig. 23:**
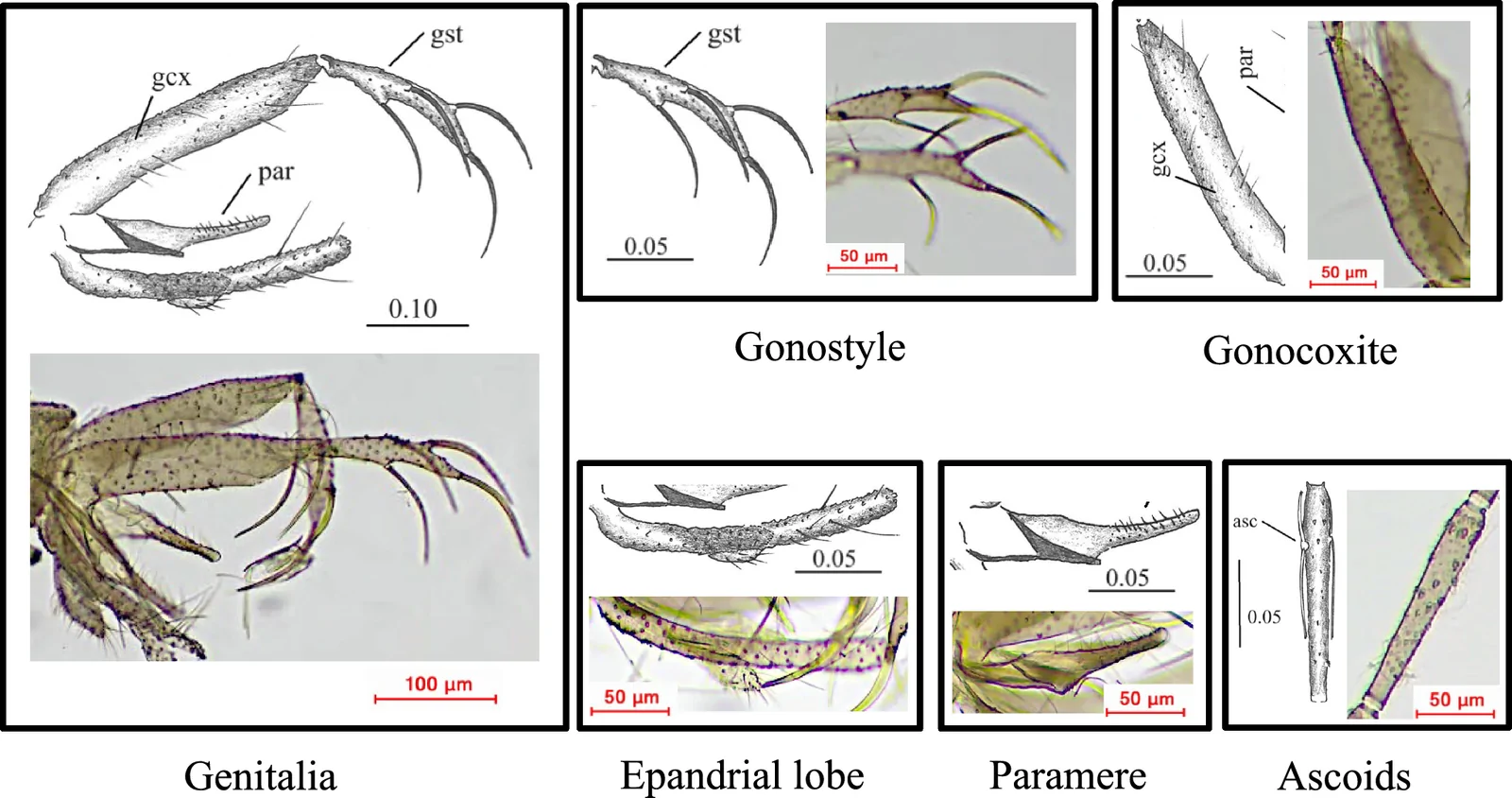
Male of *Psathyromyia shannoni*

Female (Fig. 24)

- The cibarium has four posterior teeth.
- Ascoids have a long proximal spur.
- The spermatheca is smooth and sausage-shaped. The individual spermathecal duct is about 0.5 times the length of the spermatheca, while the common duct is 2.0 times its length.

**Fig. 24:**
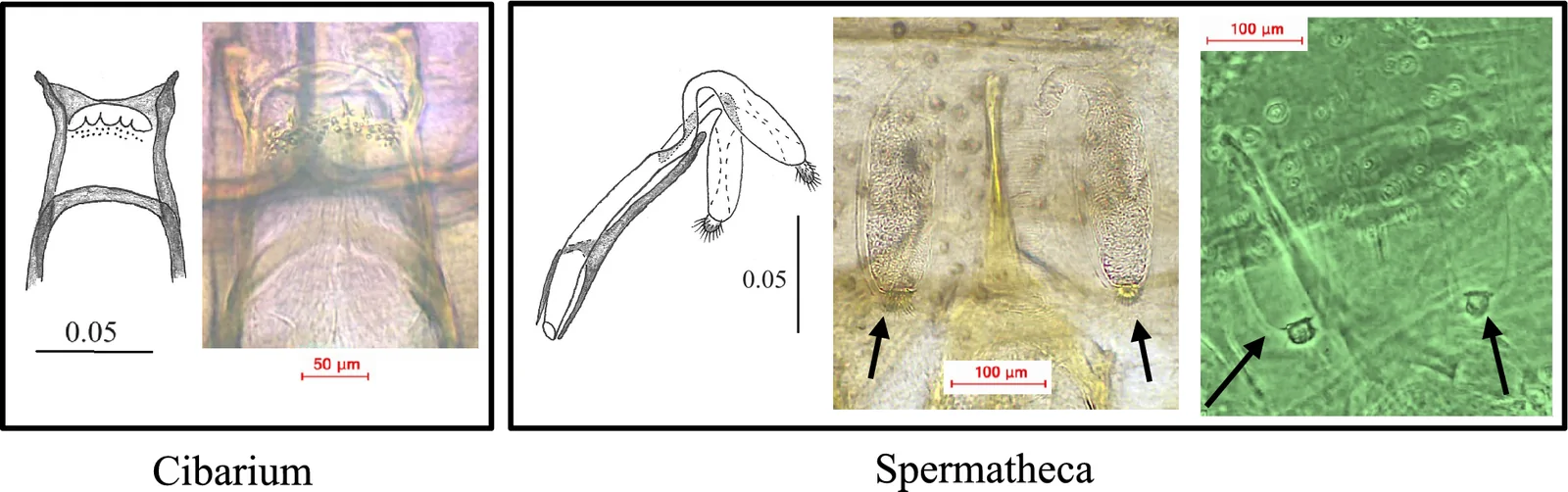
Female of *Psathyromyia shannoni*

## *Psathyromyia soccula* (Fairchild and Hertig, 1961)

Geographical distribution in Belize: this species has not previously been recorded in Belize. In the present study, a specimen was collected in the Neumaria JTA, Cayo District, representing a new country record.

Male (Fig. 25)

- Gonostyle is slender, bearing four strong spines (one apical, one upper external, one lower external, and one internal).
- Gonocoxite is elongate, with a basal tuft of long setae on the inner surface.
- Paramere is simple, elongate, slightly curved, and with an elbow in the preapical region of the ventral margin.
- Epandrial lobes are elongate and narrow, with scattered setae.

**Fig. 25:**
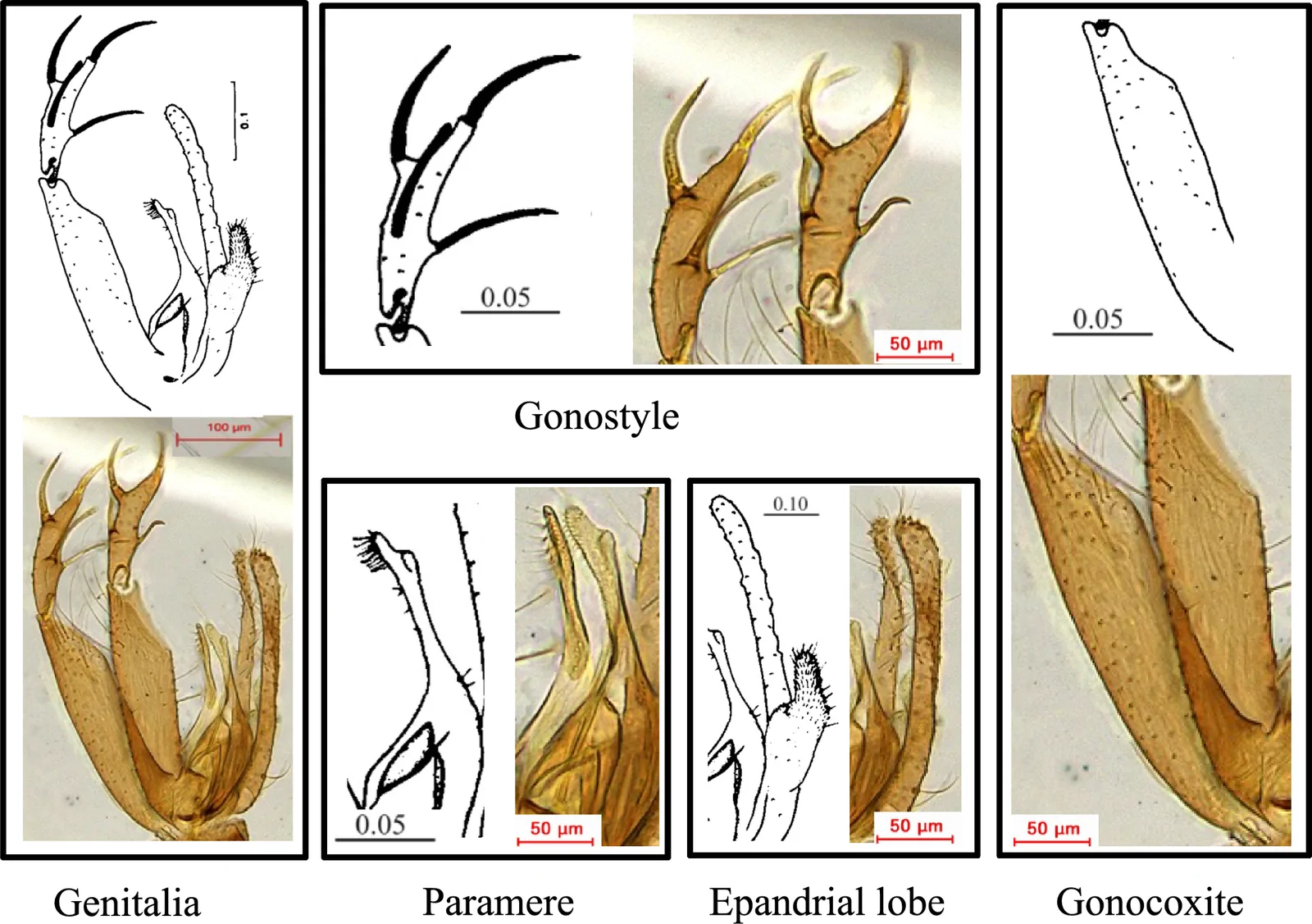
Male of *Psathyromyia soccula*

## *Psathyromyia undulata* (Fairchild & Hertig, 1950)

Geographical distribution in Belize: this species has been recorded in the Cayo District [9, 10]. In the present study, a specimen was collected only in the Cayo District (Augustine Camp JTA).

Female (Fig. 26)

- Ascoids have a long posterior spur (see *Pa. shannoni*).
- The spermatheca has about 40 thin, symmetrical annulations, with a short, rounded apical knob.
- The common spermathecal duct is 1.25 times the length of the spermatheca, while the individual spermathecal ducts are 0.75 times as long.

**Fig. 26:**
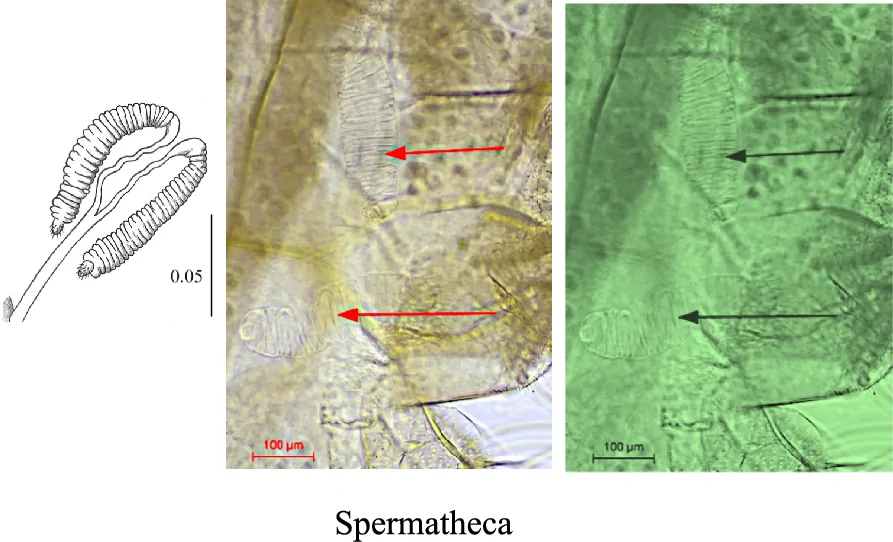
Female of *Psathyromyia undulata.* Females of this species are indistinguishable from those of *Psathyromyia cratifer*

## *Psychodopygus bispinosus* (Fairchild and Hertig, 1951)

Geographical distribution in Belize: this species has been recorded in Cayo District, Orange Walk District and Toledo District [9, 10, 15, 18]. In the present study, specimens were collected in Cayo District (Merlin and Neumaria JTAs).

Male (Fig. 27)

- The gonostyle has only two spines.
- The paramere is conical and straight.

**Fig. 27:**
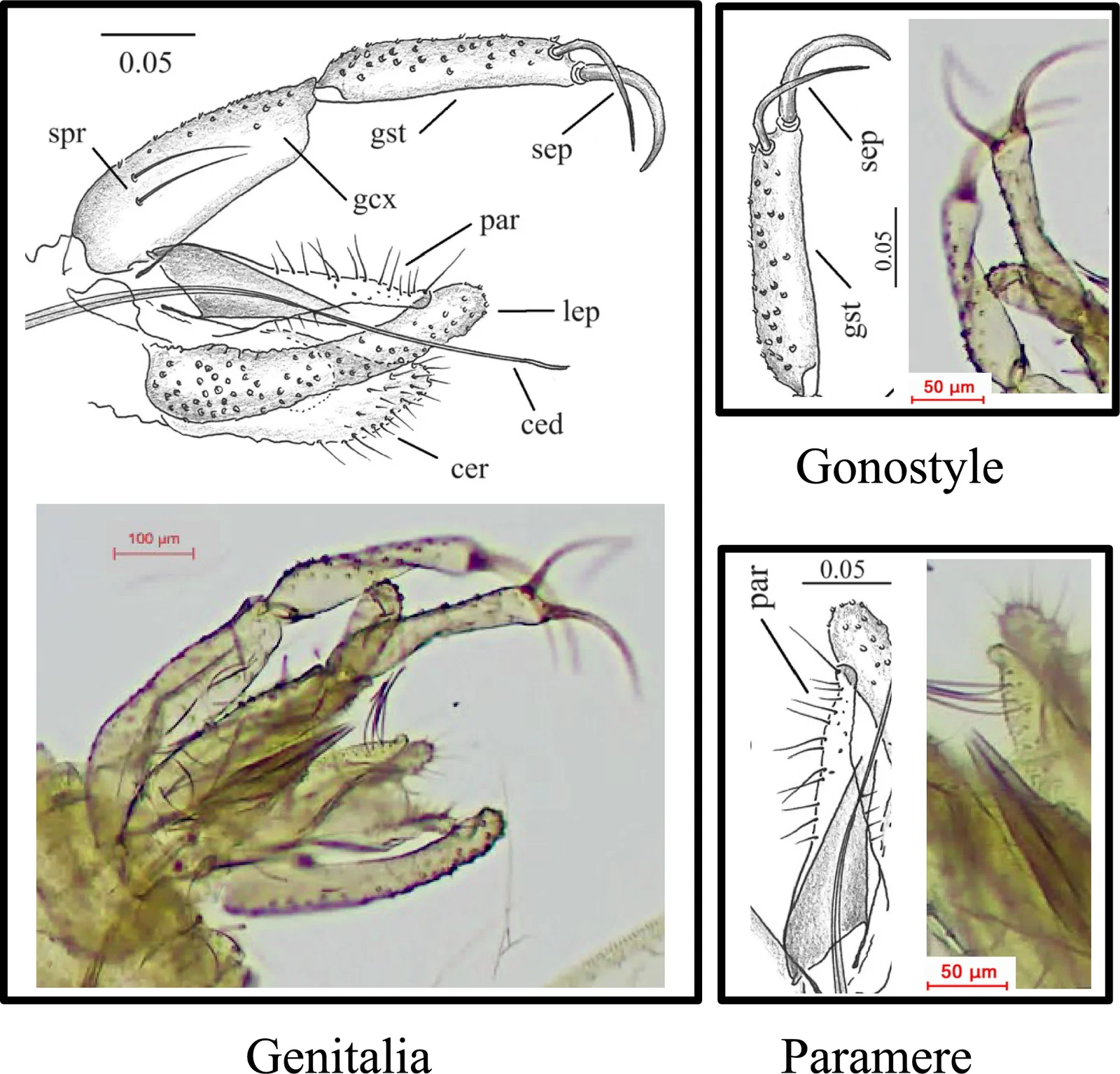
Male of *Psychodopygus bispinosus*

Female (Fig. 28)

- Palpus 5 is shorter than the combined length of palpi 3 and 4 (see *Bi. olmeca olmeca*).
- In the cibarium, a row of evenly spaced, short posterior teeth is positioned above the anterior teeth, which consist of a single row above a central cluster of teeth.
- The pigment patch is not clearly visible.
- The spermathecae are approximately 0.5 times the length of the common spermathecal duct, with the basal portion of the latter being unstriated and occupying only 0.2 of its total length.

**Fig. 28:**
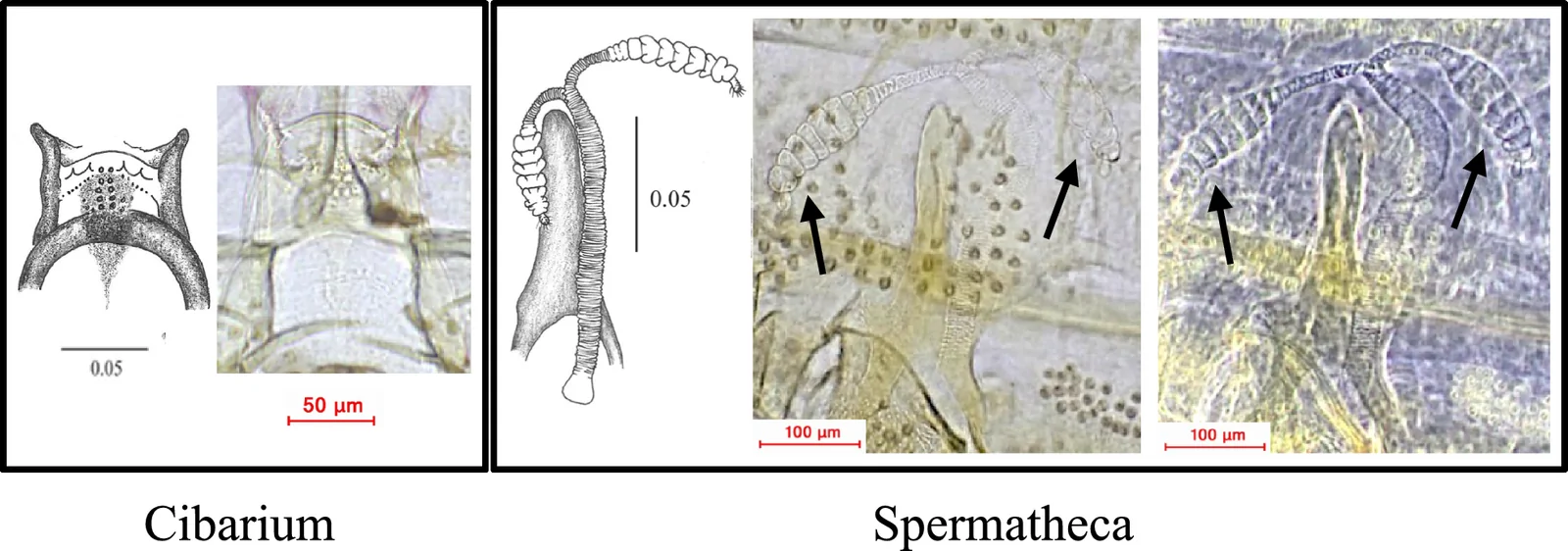
Female of *Psychodopygus bispinosus*

## *Psychodopygus geniculatus* (Mangabeira Filho, 1941)

Geographical distribution in Belize: this species has been recorded in the Cayo District [9, 10]. In the present study, specimens were collected in the Cayo District (Neumaria JTA).

Female (Fig. 29)

- Palpus 5 is shorter than 0.5 times the length of palpus 3 (see *Bi. olmeca olmeca*).
- Cibarium shows a distinct central pigmented patch and numerous anterior cibarial teeth
- The spermathecae are as long as or shorter than the individual spermathecal duct, with a symmetrical terminal knob.
- The common spermathecal duct is 4–5 times the length of the spermathecae.

**Fig. 29:**
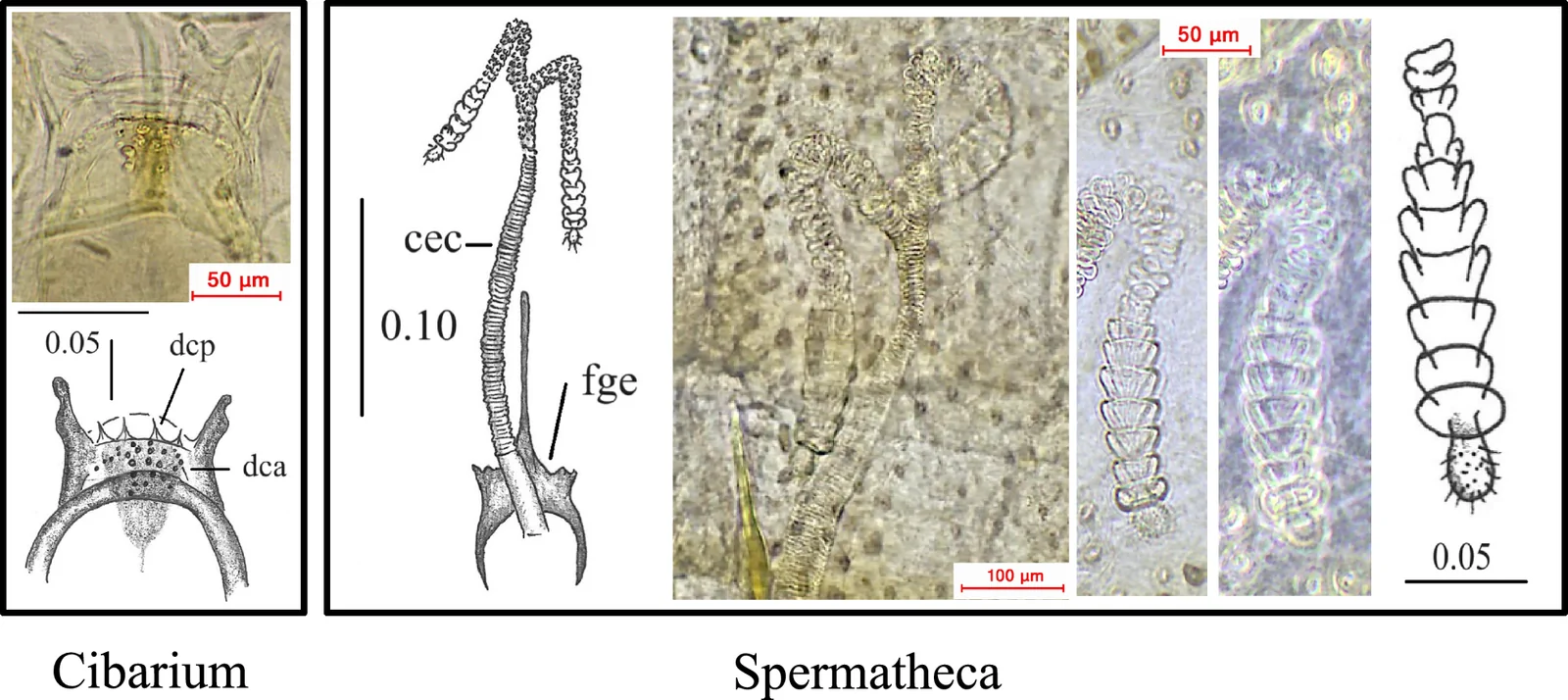
Female of *Psychodopygus geniculatus*

## *Psychodopygus panamensis* (Shannon, 1926)

Geographical distribution in Belize: this species has been recorded in the Cayo, Orange Walk, and Toledo Districts [9, 10]. In the present study, specimens were collected across multiple sites in Cayo District (Augustine Camp, Merlin, and Neumaria JTAs), Orange Walk District (Busby Camp JTA), and Belize District (Price Barracks and Manatee JTAs).

Male (Fig. 30)

- Palpus 5 is shorter than 0.5 times the length of palpus 3 (see *Bi. olmeca olmeca*).
- The gonostyle possesses four spine-shaped setae, all originating in the distal third at different levels.
- The gonocoxite has a long pre-apical seta.
- Two clusters of setae are present at the base of the paramere, with two prominent spines at its tip.
- The pre-apical seta in the paramere is delicate, while the apical seta is robust.
- The epandrial lobes are thin, long, and straight.

**Fig. 30:**
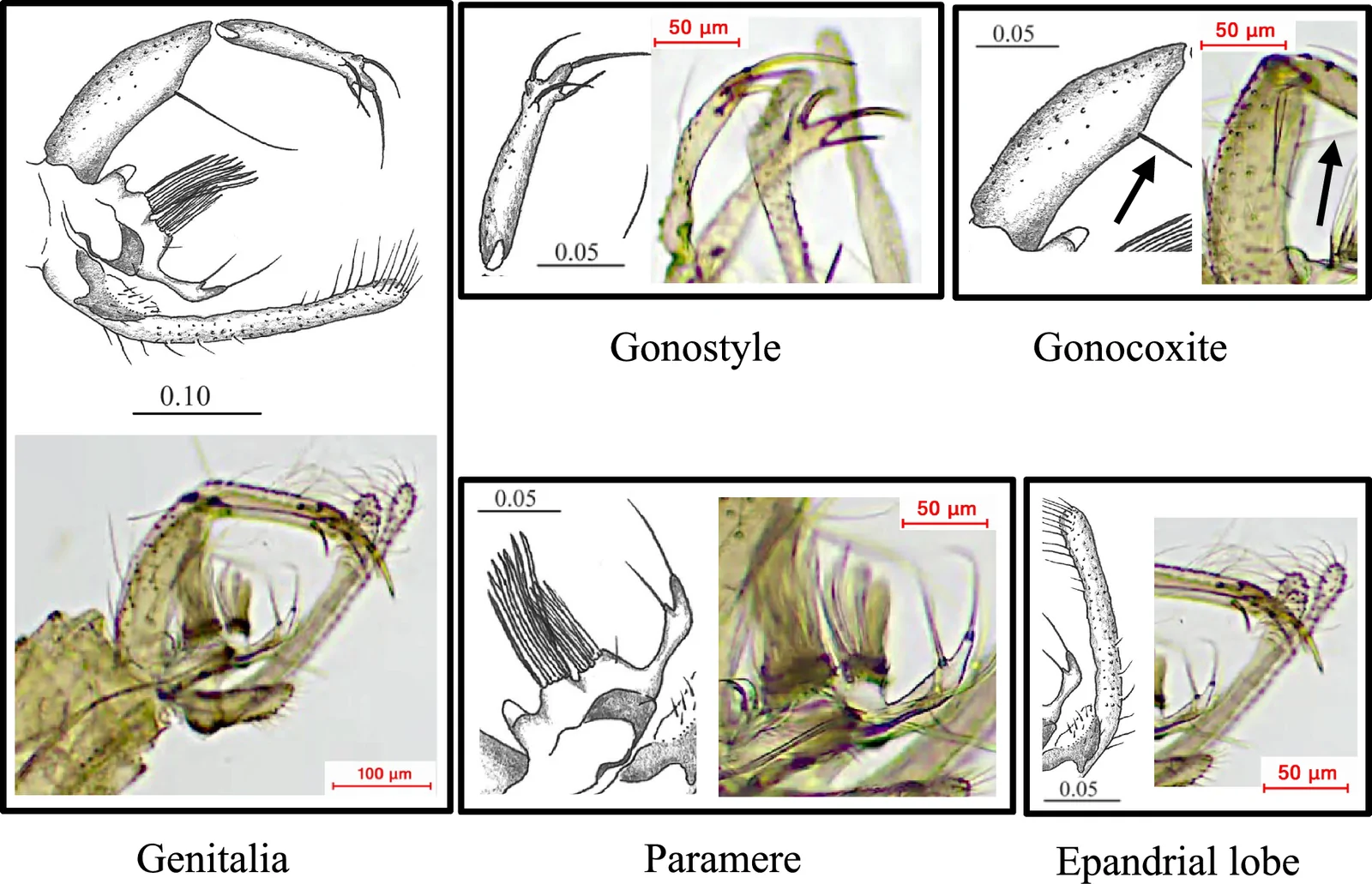
Male of *Psychodopygus panamensis*

Female (Fig. 31)

- The cibarium has elongated posterior teeth positioned above the anterior teeth, overlapped by a prominent V-shaped pigment patch that extends into the sclerotised arch and overlaps the central paired column of anterior teeth.
- The spermathecae are less than 0.4 times the length of the common spermathecal duct, with the basal half of the latter being unstriated.

**Fig. 31:**
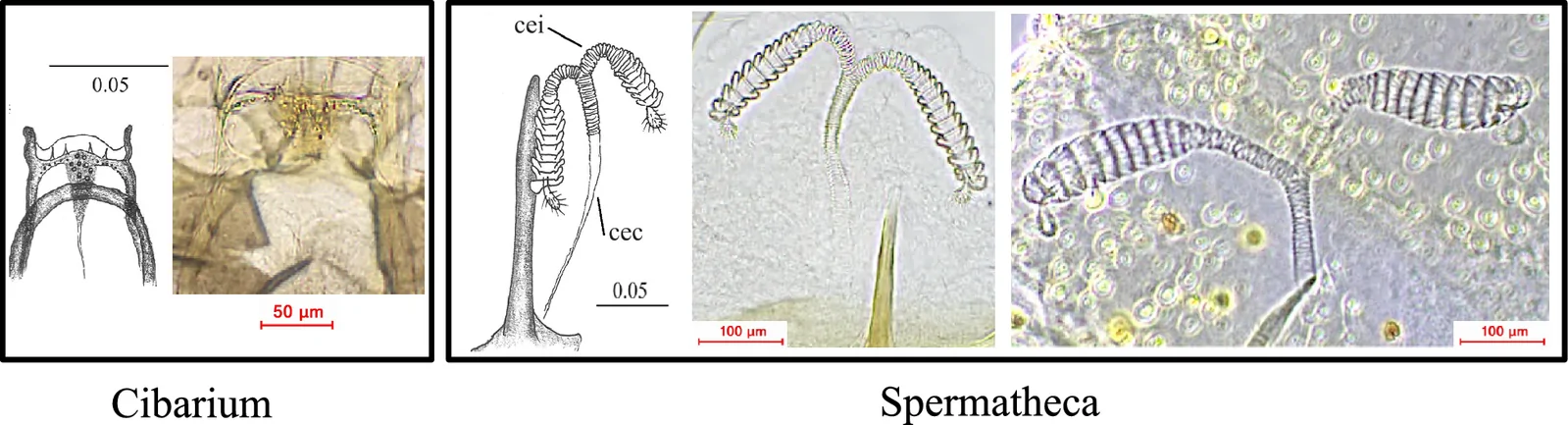
Female of *Psychodopygus panamensis*

## *Trichopygomyia triramula* (Fairchild & Hertig, 1952)

Geographical distribution in Belize: this species has been recorded in the Cayo District, Orange Walk District, and Toledo District [10]. In the present study, specimens were collected in the Cayo District (Augustine Camp and Merlin JTAs) and the Orange Walk District (Busby Camp JTA).

Male (Fig. 32)

- Palpus 5 is two times the length of palpus 3 and 4.
- The elongated gonostyle has four prominent spine-shaped setae originating at different levels.
- The thick gonocoxite lacks a setal tuft but has fine setal hairs around it.
- The paramere is coiled-shaped, featuring a cluster of setal hairs and a prominent parameral sheath at the base. It has two branches at the apex, one nearly straight and the other folded back on itself, along with a dorso-basal arm that has a curved, club-shaped apex from which robust setae originate.
- The epandrial lobes are long and slightly curved.

**Fig. 32:**
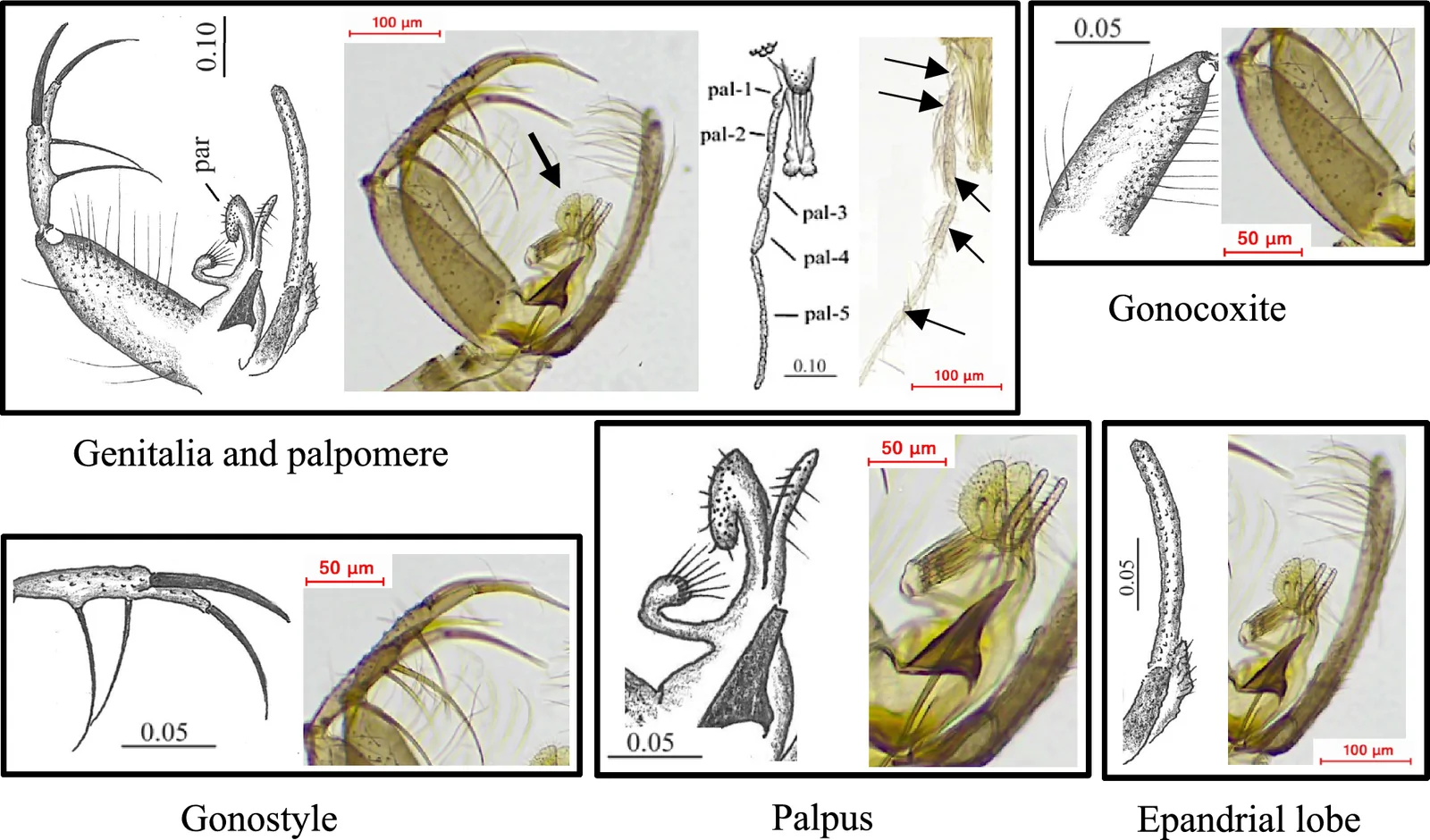
Male of *Trichopygomyia triramula*

Female (Fig. 33)

- The cibarium contains four posterior teeth arranged like a diadem, along with 1 + 1 distinct groups of lateral teeth and a transverse row of a few anterior teeth, with the central ones being larger.
- The spermathecae are globular and striated, with an apiculum that is twice as long as its width.
- The individual spermathecal ducts are approximately twice the length of the common spermathecal duct.
- The cercus is narrow at the apex, resembling a finger.

**Fig. 33:**
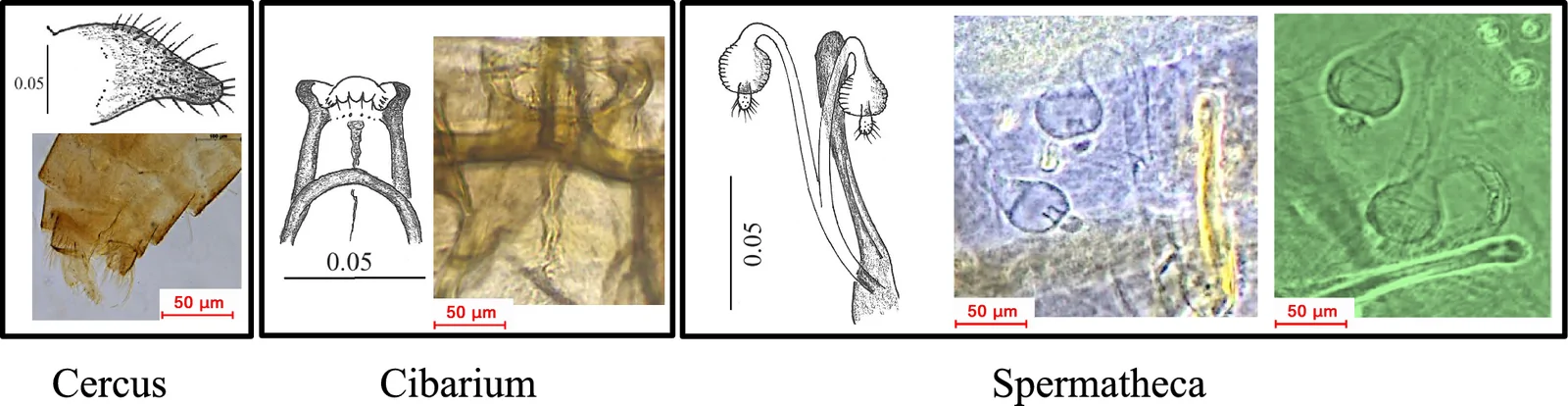
Female of *Trichopygomyia triramula*

## Discussion

This study provides a curated set of coloured photomicrographs and diagnostic notes for the 20 phlebotomine sand fly species collected during 2023–2025 in north-west and central east Belize, alongside a dichotomous key designed for use under routine light microscopy. Morphology remains the cornerstone of sand fly taxonomy and surveillance, because species delimitation in many groups relies on microscopic examination of a relatively consistent set of adult characters; most importantly, antennal ascoids, palpal segment proportions, thoracic pigmentation, wing venation, male terminalia/genitalia (e.g., gonostyle spine number/position, paramere form, gonocoxite setation), and female internal structures (e.g., spermathecal form/segmentation, cibarial armature, and pharyngeal armature) [17–19, 30]. These structures are repeatedly emphasised as the primary basis for conclusive morphological identification across regional keys and systematic treatments [31].

**Fig. 34:**
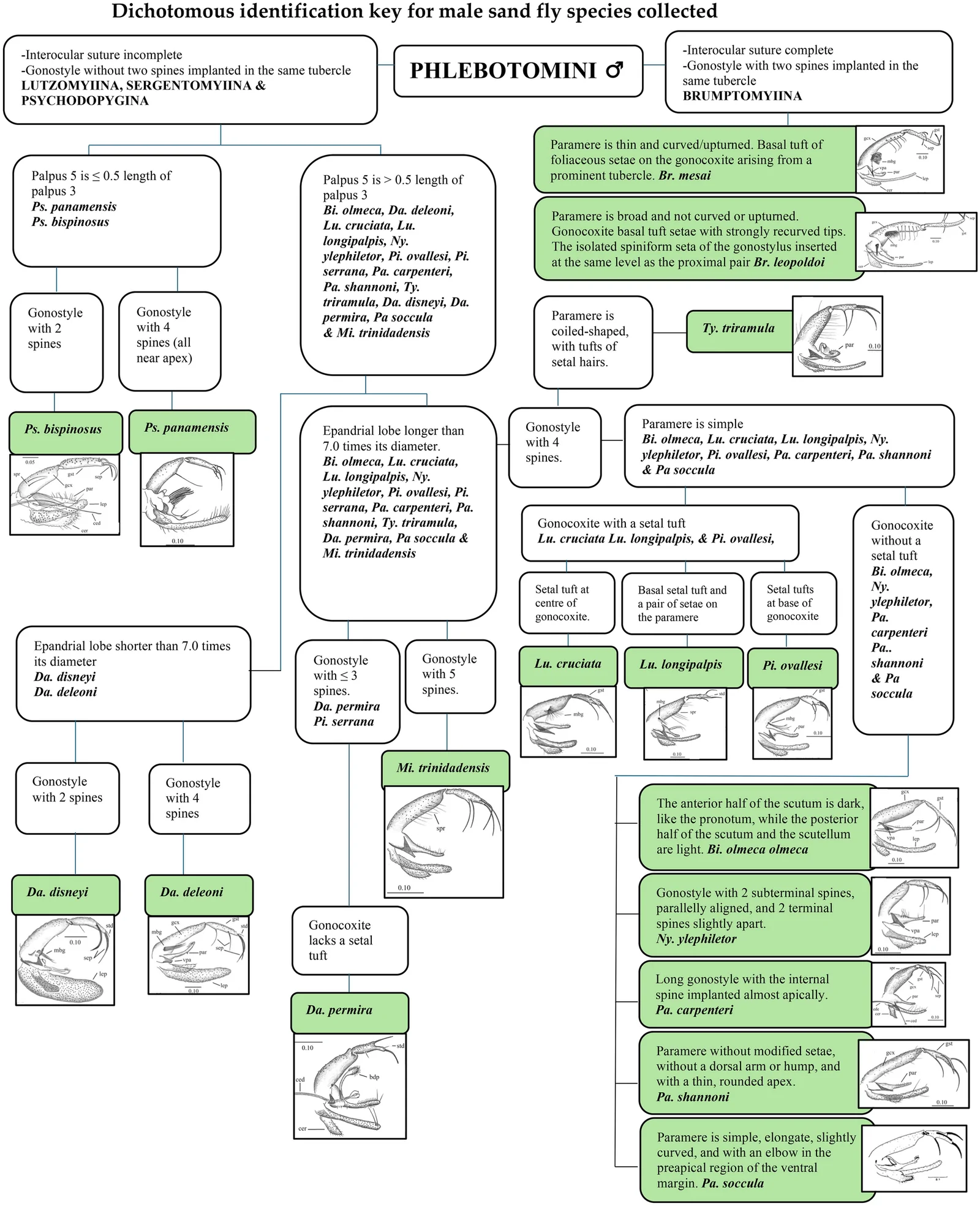
Dichotomous identification key for male sand fly species collected

**Fig. 35:**
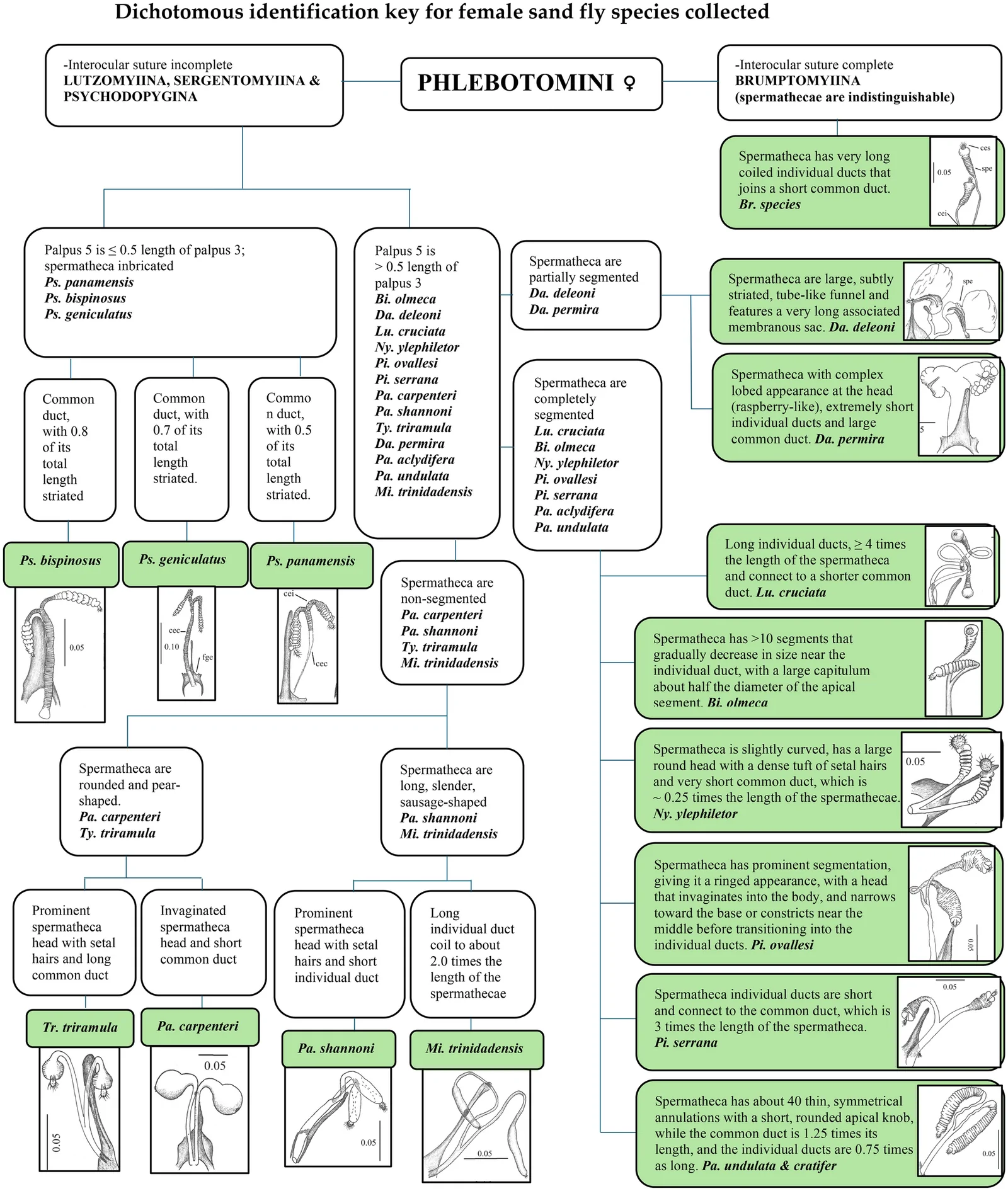
Dichotomous identification key for female sand fly species collected

A key contribution of this work is the use of colour imagery to complement traditional line drawings and greyscale figures. Although greyscale illustrations remain fundamental in sand fly systematics, recent work demonstrates that extensive pictorial resources can substantially improve the usability of morphology-based identification by clarifying subtle diagnostic features and reducing interpretive ambiguity, particularly for non-specialists and for routine surveillance workflows (Figs 34, 35). For example, Akhoundi et al. [32] recently produced a large-scale pictorial key of *Phlebotomus* sand flies in the Mediterranean and Middle Eastern regions to support consistent identification based on discriminative morphological characters, illustrating the growing role of image-rich resources in modern sand fly taxonomy.

Diagnostic reliability differs by sex and genus, and some taxa remain difficult to resolve morphologically, particularly among females (e.g., *Brumptomyia* and *Psathyromyia*; Figs. 4 and 27). This limitation is well-recognised, and morphology-based keys often rely heavily on male genital characters, because female characters can be less distinctive or more conservative in certain lineages [31]. The guide, therefore, deliberately prioritises structures that are most consistently informative at the species level (male terminalia; spermathecae and cibarium/pharynx in females) while also flagging cases, where morphology alone is insufficient for confident discrimination.

The observation of *Dampfomyia* specimens listed as *aff. permira* further highlights the importance of treating morphology as both a diagnostic and hypothesis-generating tool. Subtle but consistent differences in terminalia or spermathecal configuration may represent intraspecific variation, artefacts of slide mounting, or genuinely distinct taxa. Contemporary integrative studies increasingly show that morphologically similar or “cryptic” sand fly taxa can be resolved more robustly when morphological examination is combined with molecular approaches (e.g., COI barcoding) and formal species delimitation methods [33].

We report the first confirmed record of *Lu. longipalpis* in Belize. This species is widely distributed across the Americas and is recognised as the principal vector of *Leishmania infantum*, the causative agent of human and canine visceral leishmaniasis [34–36]. The *Lu. longipalpis* complex is well-known for its biological and population-level complexity [37–40]. Although male genital morphology (including paramere setation patterns) is central for species recognition, sibling species within the *Lu. longipalpis* complex cannot be reliably distinguished using classical morphology alone. Instead, species delimitation has relied on integrative characters, including acoustic communication signals, such as male copulation songs, together with molecular and genetic markers and differences in male sex-aggregation pheromone chemotypes [40, 41]. In this context, curated voucher deposition and image-based documentation provide an essential foundation for reproducibility and re-examination and create a practical bridge to future integrative work, where molecular tools become available.

*Psathyromyia soccula* (Fairchild & Hertig, 1961) is a Neotropical sand fly species that has been reported primarily from Central America, including Costa Rica and Panama, and is documented mainly in taxonomic catalogues and distribution checklists rather than ecological or epidemiological studies [20, 42, 43]. Within the current classification of Phlebotominae, the species belongs to the genus *Psathyromyia*, which includes several species distributed across the Neotropical region but for which biological and vectorial information remains limited [44]. The detection of a single male specimen in Belize in the present study, therefore, represents, to our knowledge, the first documented record of *Pa. soccula* in the country, extending its known geographic distribution within Central America. Given the scarcity of ecological information on this species, additional sampling and integrative taxonomic approaches will be important to confirm its presence and better characterise its distribution and potential epidemiological relevance in the region.

This pictorial guide strengthens the morphology component of sand fly surveillance capacity in Belize by: (i) standardising the visual reference for key diagnostic structures; (ii) improving interpretability of characters commonly used in dichotomous keys; and (iii) transparently flagging taxa, where morphology alone may be insufficient. In line with current practice, the next step is targeted integrative follow-up, particularly for morphologically ambiguous taxa, using barcoding and comparative morphology to confirm boundaries and support robust vector surveillance. Future surveys incorporating additional trapping methods used historically (e.g., baited oil traps, sticky traps, and Shannon traps) [9, 45, 46] may help detect additional species or clarify distribution patterns. Although molecular approaches are increasingly used for sand fly identification, morphology remains the primary tool in many endemic regions due to resource and infrastructure limitations.

## Conclusions

Traditional sand fly identification relies heavily on dichotomous keys and greyscale illustrations [18, 19]. This coloured pictorial guide complements these resources by providing visual references that may facilitate faster and more consistent identification. Similar pictorial approaches have improved identification workflows in other regions [32]. Integrating morphological resources with molecular tools represents an important next step for strengthening vector surveillance and taxonomy [47]. In settings such as Belize, where sand fly molecular information and technical expertise remain limited, accessibility to visual guides is valued for research, surveillance, and ultimately to help mitigate transmission.

## Data Availability

All data generated or analysed during this study are included in this published article and its supplementary information files.

## References

[1] Maroli M, Feliciangeli MD, Bichaud L, Charrel RN, Gradoni L. Phlebotomine sand flies and the spreading of leishmaniases and other diseases of public health concern. Med Vet Entomol. 2013;27:123–47. 10.1111/j.1365-2915.2012.01034.x. 22924419

[2] Tesh RB. The genus *Phlebovirus* and its vectors. Annu Rev Entomol. 1988;33:169–81. 10.1146/annurev.en.33.010188.001125. 2829707

[3] Jancarova M, Polanska N, Volf P, Dvorak V. The role of sand flies as vectors of viruses other than phleboviruses. J Gen Virol. 2023;104:001837. 10.1099/jgv.0.001837. 37018120

[4] Silva SP, Dilcher M, Weber F, Hufert FT, Weidmann M, Cardoso JF, et al. Genetic and biological characterization of selected Changuinola viruses (*Reoviridae*, *Orbivirus*) from Brazil. J Gen Virol. 2014;95:2251–9. 10.1099/vir.0.064691-0. 24986085 PMC5972349

[5] Arantes PVC, Pinto IS, Lee DAB, Mongruel ACB, Galati EAB, Machado RZ, et al. Molecular evidence of *Bartonella* spp. in sand flies (Diptera: Psychodidae) from the Brazilian Amazon. Acta Trop. 2025;267: 107682. 10.1016/j.actatropica.2025.107682. 40449864

[6] Lee DA, Shimabukuro PH, Brilhante AF, Arantes PV, Sanches GS, Franco EO, et al. *Bartonella* spp. in phlebotominae sand flies, Brazil. Emerg Infect Dis. 2024;30:2099–2107. 10.3201/eid3010.240397 PMC11431920 39320166

[7] Strangways-Dixon J, Lainson R. The epidemiology of dermal leishmansiasis in British Honduras Part III. The transmission of *Leishmania mexicana* to man by *Phlebotomus pessoanus*, with observations on the development of the parasite in different species of *Phlebotomus*. Trans R Soc Trop Med Hyg. 1966;60:192–207. 10.1016/0035-9203(66)90027-7. 5922608

[8] Garnham PCC, Lewis DJ. Parasites of British Honduras with special reference to leishmaniasis. Trans R Soc Trop Med Hyg. 1959;53:12–35. 10.1016/0035-9203(59)90080-X. 13635809

[9] Williams P. Observations on the phlebotomine sand flies of British Honduras. Ann Trop Med Parasitol. 1965;59:393–404. 10.1080/00034983.1965.11686325. 5862607

[10] Williams P. Phlebotomine sand flies and leishmaniasis in British Honduras (Belize). Trans R Soc Trop Med Hyg. 1970;64:317–63. 10.1016/0035-9203(70)90171-9. 5453495

[11] Williams P. On the vertical distribution of phlebotomine sandflies (*Dipt.*, *Psychodidae*) in British Honduras (Belize). Bull Entomol Res. 1970;59:637–46. 10.1017/S000748530000362X. 5450441

[12] Williams P. The form of *Lutzomyia beltrani *(Vargas & Díaz Nájera) (Diptera, Psychodidae) in Belize. Central Am Bulletin Entomol Res. 1976;65:595–9.

[13] Williams P. The phlebotomine sand flies (Diptera, Psychodidae) of caves in Belize, Central America. Bull Entomol Res. 1976;65:601–14. 10.1017/S0007485300006301.

[14] Williams P. Description of *Lutzomyia *(*Coromyia*) *disneyi*, n. sp. (Diptera: Psychodidae-Phlebotominae) from Belize, Central America. Memórias do Instituto Oswaldo Cruz. 1987:525-9.

[15] Martins AV, Williams P, Falcão AL. American sand flies (Diptera: Psychodidae, Phlebotominae). Rio de Janeiro: Academia Brasileira de Ciências; 1978.

[16] Williams P. Patterns in the geographical distribution of members of the genus *Lutzomyia *França (Diptera: Psychodidae: Phlebotominae). In: Burger JF, editor. Contributions to the knowledge of Diptera. Mem Entomol Int. 1999 14: 455–502.

[17] Killick-Kendrick R. Guide to the Identification and Geographic Distribution of *Lutzomyia *Sand Flies in Mexico, the West Indies, Central and South America (Diptera: Psychodidae). DG Young & MA Duncan. Memoirs of the American Entomological Institute no. 54. Gainesville, Florida, USA: Associated Publishers, 1994. 881 pp. US $85. ISBN 1-5666-054-2.

[18] Ibáñez-Bernal S. Notes on the Psychodidae (Diptera) of Belize: subfamilies Bruchomyiinae and Phlebotominae. Ann Entomol Soc Am. 2001;94:367–85. 10.1603/0013-8746(2001)094[0367:NOTPDO]2.0.CO;2.

[19] Galati EAB. Phlebotominae (Diptera, Psychodidae): classification, morphology and terminology of adults and identification of American taxa. In: Rangel EF, Shaw JJ, editors. Brazilian sand flies: biology, taxonomy, medical importance and control. Cham: Springer International Publishing; 2018. p. 9-212. 10.1007/978-3-319-75544-1_2

[20] Galati EAB, de Andrade AJ, Perveen F, Loyer M, Vongphayloth K, Randrianambinintsoa FJ, et al. Phlebotomine sand flies (Diptera, Psychodidae) of the world. Parasit Vectors. 2025;18:220. 10.1186/s13071-025-06748-5. 40495218 PMC12153111

[21] Strangways-Dixon J, Lainson R. Dermal leishmaniasis in British Honduras: transmission of *Leishmania brasiliensis* by *Phlebotomus* species. Br Med J. 1962;1:297–9. 10.1136/bmj.1.5274.297. 13917640 PMC1957443

[22] Lainson R, Strangways-Dixon J. *Leishmania mexicana*: the epidemiology of dermal leishmaniasis in British Honduras. Trans R Soc Trop Med Hyg. 1963;57:242–65. 10.1016/0035-9203(63)90182-2. 14047403

[23] Pech-May A, Escobedo-Ortegón FJ, Berzunza-Cruz M, Rebollar-Téllez EA. Incrimination of four sand fly species previously unrecognized as vectors of *Leishmania* parasites in Mexico. Med Vet Entomol. 2010;24:150–61. 10.1111/j.1365-2915.2010.00870.x. 20604861

[24] González C, Rebollar-Téllez EA, Ibáñez-Bernal S, Becker-Fauser I, Martínez-Meyer E, Peterson AT, et al. Current knowledge of *Leishmania* vectors in Mexico: how geographic distributions of species relate to transmission areas. Am J Trop Med Hyg. 2011;85:839–46. 10.4269/ajtmh.2011.10-0452. 22049037 PMC3205629

[25] Rowton ED, de Mata M, Rizzo N, Porter CH, Navin TR. Isolation of *Leishmania braziliensis* from *Lutzomyia ovallesi* (Diptera: Psychodidae) in Guatemala. Am J Trop Med Hyg. 1992;46:465–8. 10.4269/ajtmh.1992.46.465. 1575293

[26] Porter CH, Steurer FJ, Kreutzer RD. Isolation of *Leishmania mexicana mexicana* from *Lutzomyia ylephiletor* in Guatemala. Trans R Soc Trop Med Hyg. 1987;81:929–30. 10.1016/0035-9203(87)90355-5. 3503412

[27] Disney RHL. The discovery of the vector of *Leishmania mexicana*. Trans R Soc Trop Med Hyg. 1968;62:457. 10.1016/0035-9203(68)90103-X. 5659239

[28] Ibáñez-Bernal S. Claves actualizadas para la identificación morfológica de machos y hembras de las especies de Phlebotominae (Diptera: Psychodidae) conocidas en México. Acta Zool Mex. 2024;40:1–55. 10.21829/azm.2024.4012693.

[29] Galati EAB, Galvis-Ovallos F, Lawyer P, Léger N, Depaquit J. An illustrated guide for characters and terminology used in descriptions of Phlebotominae (Diptera, Psychodidae). Parasite. 2017;24:26. 10.1051/parasite/2017027. 28730992 PMC5520390

[30] Abonnenc E. Les phlébotomes de la région éthiopienne (Diptera, Psychodidae). Cah ORSTOM Sér Entomol Méd Parasitol. 1972;55:1–239.

[31] Dantas-Torres F, Tarallo VD, Otranto D. Morphological keys for the identification of Italian phlebotomine sand flies (Diptera: Psychodidae: Phlebotominae). Parasit Vectors. 2014;7:479. 10.1186/s13071-014-0479-5. 25323537 PMC4203899

[32] Akhoundi M, Maroli M, Gradoni L, Izri A, Dedet JP, Votýpka J, et al. A pictorial identification key for Mediterranean and Middle Eastern *Phlebotomus* sand flies. Sci Rep. 2025;15:7492. 10.1038/s41598-024-77815-7. 40032876 PMC11876346

[33] Soomro S, Tuangpermsub S, Ngamprasertwong T, Kaewthamasorn M. An integrative taxonomic approach reveals two putatively novel species of phlebotomine sand fly (Diptera: Psychodidae) in Thailand. Parasit Vectors. 2025;18:1. 10.1186/s13071-024-06640-8. 39762896 PMC11702185

[34] Ibáñez-Bernal S, Durán-Luz J. An actualized catalogue of the Psychodidae (Diptera) of Mexico and their known distribution by state. Zootaxa. 2022;5104:3. 10.11646/zootaxa.5104.3.2. 35391032

[35] Mejía Á, Matamoros G, Fontecha G, Sosa-Ochoa W. Bionomic aspects of *Lutzomyia evansi* and *Lutzomyia longipalpis*, proven vectors of *Leishmania infantum* in an endemic area of non-ulcerative cutaneous leishmaniasis in Honduras. Parasit Vectors. 2018;11:15. 10.1186/s13071-017-2605-7. 29304878 PMC5756426

[36] Cecílio P, Pires ACAM, Valenzuela JG, Pimenta PFP, Cordeiro-da-Silva A, Secundino NFC, et al. Exploring *Lutzomyia longipalpis* sand fly vector competence for *Leishmania major* parasites. J Infect Dis. 2020;222:1199–203. 10.1093/infdis/jiaa203. 32328656 PMC7459136

[37] Lanzaro GC, Ostrovska K, Herrero MV, Lawyer PG, Warburg A. *Lutzomyia longipalpis* is a species complex: genetic divergence and interspecific hybrid sterility among three populations. Am J Trop Med Hyg. 1993;48:839–47. 10.4269/ajtmh.1993.48.839. 8333579

[38] Bauzer LGSR, Souza NA, Maingon RDC, Peixoto AA. *Lutzomyia longipalpis* in Brazil: a complex or a single species? A mini-review. Mem Inst Oswaldo Cruz. 2007;102:1–12. 10.1590/S0074-02762007000100001. 17293992

[39] Vigoder FM, Souza NA, Brazil RP, Bruno RV, Costa PL, Ritchie MG, et al. Phenotypic differentiation in love song traits among sibling species of the *Lutzomyia longipalpis* complex in Brazil. Parasites Vectors. 2015;8:290. 10.1186/s13071-015-0900-8. 26017472 PMC4456791

[40] Sousa-Paula LC. The *Lutzomyia longipalpis* complex: what’s next? Trends Parasitol. 2025;41:963–73. 10.1016/j.pt.2025.08.007. 40885645 PMC13154714

[41] Spiegel CN, Dias DBS, Araki AS, Hamilton JGC, Brazil RP, Jones TM. The *Lutzomyia longipalpis* complex: a brief natural history of aggregation-sex pheromone communication. Parasites Vectors. 2016;9:580. 10.1186/s13071-016-1866-x. 27842601 PMC5109651

[42] Fairchild GB, Hertig M. Notes on the *Phlebotomus* of Panama IX. Descriptions of seven new species. Ann Entomol Soc Am. 1952;45:505–28. 10.1093/aesa/45.4.505.

[43] Galati EAB, de Andrade AJ, Perveen F, Loyer M, Vongphayloth K, Randrianambinintsoa FJ, et al. Phlebotomine sand flies (Diptera, Psychodidae) of the world. Parasites Vectors. 2025;18:220. 10.1186/s13071-025-06748-5. 40495218 PMC12153111

[44] Adams ZJO, Shimabukuro PHF. A cybercatalogue of American sand fly types (Diptera, Psychodidae, Phlebotominae) deposited at the Natural History Museum, London. Biodivers Data J. 2018;6:e24484. 10.3897/BDJ.6.e24484. PMC6055547 30046275

[45] Shaw JJ, de Rosa AT, Cruz AC, Vasconcelos PFC. Brazilian phlebotomines as hosts and vectors of viruses, bacteria, fungi, protozoa (excluding those belonging to the genus *Leishmania*) and nematodes. In: Rangel EF, Shaw JJ, editors. Brazilian sand flies: biology, taxonomy, medical importance and control. Cham: Springer International Publishing; 2018. p. 417-41. 10.1007/978-3-319-75544-1_9

[46] Disney RHL. A trap for phlebotomine sand flies attracted to rats. Bull Entomol Res. 1966;56:445–51. 10.1017/S0007485300056510. 5945565

[47] Williams P, Lewis DJ, Garnham PCC. On dermal leishmaniasis in British Honduras. Trans R Soc Trop Med Hyg. 1965;59:64–71. 10.1016/0035-9203(65)90140-9. 14287622

